# Wind-Turbine and Wind-Farm Flows: A Review

**DOI:** 10.1007/s10546-019-00473-0

**Published:** 2019-09-20

**Authors:** Fernando Porté-Agel, Majid Bastankhah, Sina Shamsoddin

**Affiliations:** 1 Wind Engineering and Renewable Energy Laboratory (WIRE), École Polytechnique Fédérale de Lausanne (EPFL), EPFL-ENAC-IIE-WIRE, 1015 Lausanne, Switzerland; 2 Present Address: Department of Engineering, Durham University, Durham, DH1 3LE UK

**Keywords:** Atmospheric boundary layer, Turbulence, Wind energy, Wind-farm flow, Wind-turbine wake

## Abstract

Wind energy, together with other renewable energy sources, are expected to grow substantially in the coming decades and play a key role in mitigating climate change and achieving energy sustainability. One of the main challenges in optimizing the design, operation, control, and grid integration of wind farms is the prediction of their performance, owing to the complex multiscale two-way interactions between wind farms and the turbulent atmospheric boundary layer (ABL). From a fluid mechanical perspective, these interactions are complicated by the high Reynolds number of the ABL flow, its inherent unsteadiness due to the diurnal cycle and synoptic-forcing variability, the ubiquitous nature of thermal effects, and the heterogeneity of the terrain. Particularly important is the effect of ABL turbulence on wind-turbine wake flows and their superposition, as they are responsible for considerable turbine power losses and fatigue loads in wind farms. These flow interactions affect, in turn, the structure of the ABL and the turbulent fluxes of momentum and scalars. This review summarizes recent experimental, computational, and theoretical research efforts that have contributed to improving our understanding and ability to predict the interactions of ABL flow with wind turbines and wind farms.

## Introduction

Renewable energy is expected to play a major role in meeting future world energy needs while mitigating climate change and environmental pollution. While world energy demand continues to increase at an average annual rate of about 2%, most of that demand (around 80%) is being met by fossil fuels (IEA 2018), with the well-known negative impacts on the environment and climate. This, together with the growing safety concerns surrounding nuclear energy, has led many countries to set ambitious strategic targets for renewable energies with low greenhouse gas and pollutant emissions, including wind energy (a summary of those renewable energy targets can be found in REN21 (2018)). If those targets are to be met, the total amount of installed wind-energy capacity should increase substantially in the coming decades (a review of projections of future global growth of renewable energies is provided by REN21 (2017)). Achieving that growth will necessarily require the design and installation of new large wind farms and the upgrade of existing ones in regions of high wind-energy potential.

Since the seminal works of Betz (1920) and Joukowsky (1920), substantial research efforts have been made in the field of wind-turbine aerodynamics, and particularly in the optimization of horizontal-axis wind turbine (HAWT) rotors. Glauert (1935) achieved a major breakthrough when he formulated the blade-element momentum (BEM) theory. This theory, which was later extended with many ‘engineering rules’, constitutes the basis for all rotor design optimization codes used in the industry today (see reviews by Sørensen 2011a, 2016, and references therein). These advances in wind-turbine aerodynamics have led to modern HAWTs achieving power coefficients (based on aerodynamic efficiency) of around 0.5, which is fairly close, given the unavoidable aerodynamic losses, to the maximum theoretical Betz–Joukowsky limit of 0.593 (Betz 1920; Joukowsky 1920). Moreover, reasonably accurate predictions of the performance of those turbines can be achieved using those theories if the incoming flow is known a priori. In contrast, the prediction of wind-turbine and wind-farm performance under real conditions remains an elusive target and one of the main challenges in optimizing the layout, operation, and control of wind farms. This is due to the complex interactions between wind turbines and the atmospheric boundary layer (ABL), which is highly turbulent, non-stationary (owing to the effects of the diurnal cycle and synoptic-forcing variability), modulated by ubiquitous thermal effects, and often heterogeneous (due to the effects of topography and land-surface heterogeneity). Moreover, inside wind farms, the turbulent wake flows that form downwind of the turbines are responsible for substantial power losses, due to the reduced wind speed in the wakes, as well as increased fatigue loads and associated maintenance costs, due to the augmented turbulence levels (e.g., see reviews by Vermeer et al. 2003; Sanderse et al. 2011; Stevens and Meneveau 2017, and references therein). Consequently, any improvements in the understanding and prediction of the interaction of the ABL flow with wind turbines and wind farms can potentially help increase the economic feasibility of wind-energy projects.

**Fig. 1:**
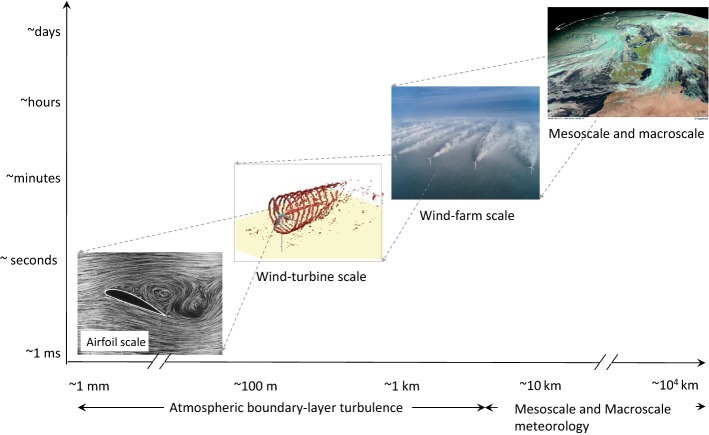
Schematic illustrating the wide range of flow scales relevant to wind energy: from the turbine blade scale to the meteorological mesoscale and macroscale

There is a wide range of atmospheric flow scales that affect wind farms, as illustrated in Fig. 1. Macroscale and mesoscale weather phenomena are responsible for the variability of flow in the free atmosphere at horizontal length scales larger than about 2000 km, and in the range of 2–2000 km, respectively (Orlanski 1975). This variability in large-scale atmospheric motions, combined with the modulating effects of the Coriolis force, the aerodynamic forces on land or sea surfaces, plant canopies, buildings, topography, and wind turbines, as well as atmospheric stability, regulate the structure and evolution of the ABL inside and around wind farms. The continuous range of turbulence scales in the ABL, spanning from the integral scale (on the order of 1 km and 100 s) down to the Kolmogorov scale (on the order of 1 mm and 1 ms), plays a key role in the adjustment of the ABL around wind turbines and farms (including turbine wakes) and, ultimately, on their performance. The multi-scale nature of atmospheric turbulence over such a wide range of scales makes the modelling and measurement of the ABL flow and its two-way interaction with wind farms particularly challenging.

A variety of analytical, computational, and experimental approaches have been used in recent years to study the interaction of turbulent ABL flow with wind turbines and wind farms. Some of the most relevant are briefly introduced below:

- *Analytical modelling*: Several simple analytical models have been proposed for the prediction of the average velocity deficit in wind-turbine wakes (e.g., Jensen 1983; Frandsen et al. 2006; Bastankhah and Porté-Agel 2014b). Even though they are necessarily less accurate than more sophisticated turbulence-resolving numerical simulation tools, their simplicity and low computational cost ($\sim$ $10^{{-3}}$ CPU hours per simulation) makes them the preferred choice for the purposes of optimizing the layout and control of wind farms over flat terrain (e.g., offshore). This is because optimization techniques, such as genetic algorithms, particle swarm optimizationm, or sequential quadratic programming, need the simulation of thousands of cases encompassing the combination of multiple wind conditions (directions and magnitudes), as well as wind-farm configurations and/or control strategies. Analytical models have also been developed to predict the vertical distribution of the mean area-averaged wind speed in infinite wind farms (e.g., Frandsen 1992; Calaf et al. 2010; Yang et al. 2012; Abkar and Porté-Agel 2013) and also to parametrize the effect of wind farms in weather models (e.g., Baidya Roy et al. 2004; Blahak et al. 2010; Fitch et al. 2012; Abkar and Porté-Agel 2015b). Compared to other simple models that have a more empirical basis, analytical models have the added value of providing fundamental insight into the physics, as their derivation relies on the application of the basic equations governing the conservation of flow properties (e.g., mass, momentum, and energy).
- *Computational fluid dynamics (CFD)*: The Reynolds-averaged Navier–Stokes (RANS) technique has been extensively used to study wind-turbine and wind-farm flows (e.g., see reviews by Vermeer et al. 2003; Sanderse et al. 2011). With the fast growth of computational power, important progress has been made in the last decade in the development, validation, and application of turbulence-resolving CFD tools, and particularly large-eddy simulation (LES) for wind-energy applications (e.g., see the review by Mehta et al. 2014). Unlike RANS and other reduced-order models (e.g., linearized Navier–Stokes solvers), where all the scales of the turbulence are parametrized, LES only requires the parametrization of the smallest (subgrid) scales, while the larger and more energetic scales are explicitly resolved. Nonetheless, LES of complex turbulent flows is known to be sensitive to the parametrization of subgrid-scale turbulent fluxes and subgrid-scale forces, including turbine-induced forces. In spite of this and the relatively high computational cost of LES ($\sim$ $10^{{3}}-10^{{4}}$ CPU hours per simulation), recent validation studies have demonstrated that, with the appropriate parametrizations, LES can yield accurate simulations of turbulent boundary-layer flow around wind turbines and wind farms (e.g., Wu and Porté-Agel 2011, 2013; Yang et al. 2014c; Xie and Archer 2015; Draper et al. 2016; Stevens et al. 2018).
- *Wind-tunnel experiments*: Numerous wind-tunnel experiments have been carried out in the last decades to study airflow around wind turbines in freestream (uniform and nearly laminar) inflow. An extensive review of this literature is given by Vermeer et al. (2003). During the last few years, wind-tunnel experiments have also been performed to study the interaction between turbulent boundary-layer flows and wind turbines or farms (e.g., Chamorro and Porté-Agel 2009, 2011; Cal et al. 2010; Lebron et al. 2012; Aubrun et al. 2013; Tian et al. 2013; Hancock and Pascheke 2014; Hamilton et al. 2015; Li et al. 2016; Bastankhah and Porté-Agel 2017c; Hyvärinen et al. 2018). These experiments have provided valuable information on the flow structure of turbine wakes in boundary-layer flows, which exhibit important differences with respect to those in freestream flows. They have also provided unique datasets for the validation of analytical models and CFD models, such as RANS and LES models.
- *Field experiments*: Recent work has attempted to overcome the difficulties inherent in measuring turbulent flow around wind turbines in the field. For example, some early field experiments were carried out using anemometers mounted on meteorological towers to characterize wind-turbine wake flows (e.g., Cleijne 1992, 1993; Duckworth and Barthelmie 2008). More recently, the application of remote sensing technologies, such as scanning wind lidars (e.g., Käsler et al. 2010; Iungo et al. 2013b; Aitken et al. 2014a; Aitken and Lundquist 2014; Banta et al. 2015; Vollmer et al. 2015; Machefaux et al. 2015; Bodini et al. 2017; Fuertes et al. 2018) and radars (e.g., Hirth and Schroeder 2013; Hirth et al. 2015), is providing new insights into the effect of atmospheric turbulence on the structure and dynamics of the flow around wind turbines and wind farms, as well as valuable datasets for testing numerical models.

The present article reviews recent theoretical, experimental, and computational research on wind-turbine and wind-farm flows, with emphasis on turbine wakes and their interaction with the ABL. It is organized as follows: Sect. 2 focuses on the flow around stand-alone wind turbines, while the flow within and around wind farms over flat terrain is discussed in Sect. 3. Two topics that are relatively under-explored, but are receiving increasing levels of attention, relate to topographical effects and vertical-axis wind turbines (VAWTs), which are discussed in Sects. 4 and 5, respectively. Finally, a summary and future perspectives are given in Sects. 6 and 7. Particular emphasis is placed on identifying knowledge gaps and open scientific questions that present opportunities for future research.

**Fig. 2:**
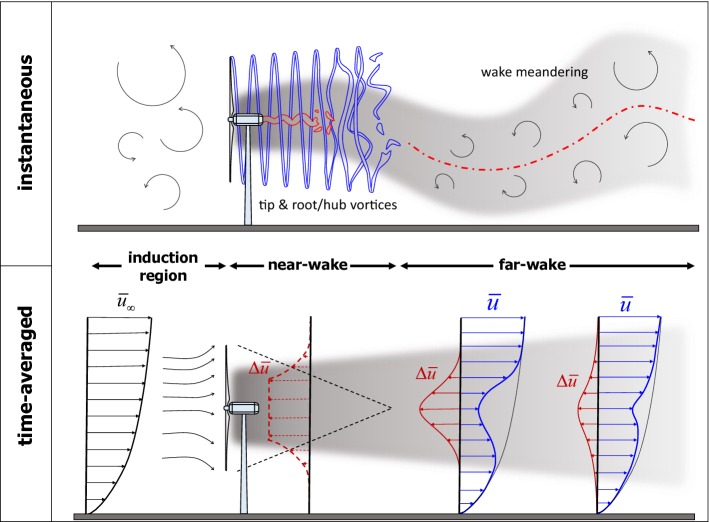
Schematic figure showing the flow regions resulting from the interaction of a wind turbine and incoming turbulent boundary layer. Depicted are the most characteristic instantaneous (top) and time-averaged (bottom) flow features

## Flow Around a Wind Turbine

The presence of a wind turbine affects the airflow both upwind and downwind of the turbine (Wilson et al. 1976; Spera 1994; Burton et al. 1995). The upwind region affected by the turbine is called the *induction region*. Prior studies (e.g., Medici et al. 2011; Simley et al. 2016) have shown that the main impact of the turbine on that region is a reduction in wind speed, which can be estimated acceptably with the following simple relationship based on the vortex sheet theory (Medici et al. 2011),

1 $$\begin{aligned} \frac{{\bar{u}}}{{\bar{u}}_\infty }=1-a\left( 1+\frac{2x}{d}\left( 1+ \left( \frac{2x}{d}\right) ^2\right) ^{-0.5}\right) , \end{aligned}$$

where *u* is the streamwise velocity component along the rotor axis (the overbar denotes time averaging), *x* is the streamwise position (being zero at the turbine and negative upwind), $u_\infty$ is the streamwise velocity component far upwind, *d* is the rotor diameter, and *a* is the rotor induction factor.

The region downwind of the turbine is called the *wake*. The wind-turbine wake itself is generally divided into two regions (Vermeer et al. 2003): (i) the region immediately downwind of the turbine with a length of 2–4 rotor diameters, called the *near-wake*, and (ii) the region further downstream, called the *far-wake*. Figure 2 shows a schematic of the different regions affected by the presence of the wind turbine.

The near-wake is directly influenced by the presence of the wind turbine, so characteristics of the turbine, such as the blade profile, hub and nacelle geometry, can affect the flow field in this region (Crespo et al. 1999b). As a result, the near-wake is characterized by highly complex, three-dimensional (3D), and heterogeneous flow distribution. In contrast, the far-wake region is less influenced by detailed features of the wind turbine. Instead, global wind-turbine parameters, such as the thrust and power coefficient, and incoming flow conditions, are likely enough to predict the mean flow distribution in this region. In the following, we provide an overview of the aerodynamic research on wakes (both near- and far-wake regions) of single turbines in horizontally-homogeneous boundary layers.

**Fig. 3:**
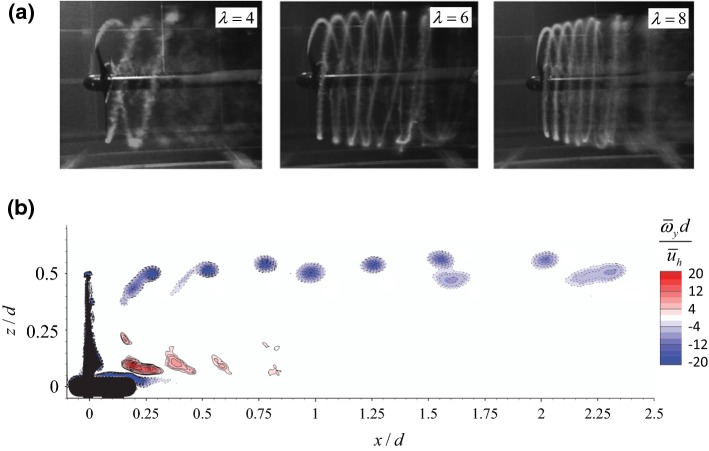
**a** Flow visualizations of the 3D helical vortex structures behind a turbine rotor for different values of tip-speed ratio $\lambda$ (figure reprinted from Okulov et al. (2014) with permission of Cambridge University Press), **b** phase-averaged contours of the out-of-plane vorticity for the wake of a turbine, obtained with particle-image velocimetry measurements. Both tip and root vortices can be seen in the figure, and the pairing of tip vortices is evident as they move downstream (figure reprinted from Sherry et al. (2013a) with permission of AIP Publishing)

### Near-Wake

#### Tip and Root Vortices

The most striking features of turbine near-wakes are perhaps the periodic helicoidal vortex structures shedding from the tip and the root of the rotor blades (Fig. 2). The presence of tip and root vortices in the near-wake of wind turbines has been widely demonstrated in the literature (see Fig. 3, for instance). These vortex structures are caused by the difference in pressure between the pressure and suction sides of the rotor blade (Andersen et al. 2013). Consequently, their shedding frequency is three times of the rotor rotational frequency for a three-bladed HAWT. While the helix pitch (i.e., the streamwise distance between two consecutive vortices) of tip vortices is evidently greater than the pitch of the root vortices, both decrease with the increase of tip-speed ratio (i.e., the ratio between the velocity of the blade tip to that of the unperturbed incoming flow at hub height) (Whale et al. 2000; Hu et al. 2012; Sherry et al. 2013b). The evolution and stability of tip and root vortices have received extensive attention in the literature both numerically (e.g., Ivanell et al. 2010; Lu and Porté-Agel 2011; Sarmast et al. 2014, 2016; Mirocha et al. 2015; Nilsson et al. 2015; Premaratne and Hu 2017; Tabib et al. 2017) and experimentally (e.g., Whale et al. 2000; Grant and Parkin 2000; Zhang et al. 2012, 2013b; Chamorro et al. 2013b; Jin et al. 2014; Lignarolo et al. 2016; Yang et al. 2016; Wei et al. 2017). The main focus has been given to the study of tip vortices as they are more persistent (Sherry et al. 2013b). Moreover, tip vortices can reduce flow entrainment in the near wake by separating this region from the outer flow (Lignarolo et al. 2014). Therefore, it is of great interest to understand the underlying mechanisms that lead to the evolution and breakdown of tip vortices. To this end, several wind-tunnel studies have been performed based on high-resolution particle-image velocimetry measurements (both phase-locked and free-run) to visualize the tip vortices at different locations and time instants. These studies reported that tip vortices have some random fluctuations around their statistically-averaged positions. These random motions are referred to as *vortex wandering* or *vortex jittering* (Heyes et al. 2004), and their amplitude increases with the vortex age (Dobrev et al. 2008; Hu et al. 2012; Sherry et al. 2013a) and the incoming turbulence intensity (Beresh et al. 2010).

Different mechanisms have been proposed to be responsible for the breakdown of helical vortex filaments (Widnall 1972; Sørensen 2011b). The mutual inductance instability is, however, considered as the dominant mode of instability for helical vortex filaments when the helix pitch decreases beyond a certain limit (Widnall 1972; Felli et al. 2011). The mutual inductance instability results in the pairing of tip vortices and ultimately their breakdown (Odemark and Fransson 2013; Sarmast et al. 2014; Eriksen and Krogstad 2017). The decrease of helix pitch intensifies the mutual inductance instability, so the breakdown of tip vortices occurs faster at higher tip-speed ratios (Sørensen et al. 2015). It is also important to note that, under turbulent boundary-layer inflow conditions, the lifetime of tip vortices is significantly reduced due to the relatively high turbulence intensity and wind shear (Lu and Porté-Agel 2011; Zhang et al. 2012, 2013b; Hong et al. 2014; Khan et al. 2017).

#### Hub Vortex

The presence of the so-called hub vortex, a vortical structure located at the central part of the near-wake and elongated in the streamwise direction, has recently received some attention. Several wind-tunnel and numerical studies (e.g., Felli et al. 2011; Iungo et al. 2013a; Viola et al. 2014; Ashton et al. 2016) have shown that the hub vortex is characterized by a single-helix counter-winding instability, which interacts with the tip-vortex layer (e.g., Okulov and Sørensen 2007; Kang et al. 2014; Howard et al. 2015). This helical vortex structure induces periodic motions in the central part of the near-wake. Similar periodic motions in the central part of the near-wake have been also associated to vortex shedding (e.g., Medici and Alfredsson 2006), commonly seen behind bluff bodies (e.g., cylinders). It is a common practice to describe the frequency of periodic oscillations by the dimensionless Strouhal number *St*, which is given by $St=fd/{\bar{u}}_h$, where *f* is the oscillation frequency, *d* is the rotor diameter, and ${\bar{u}}_h$ is the mean incoming wind speed at hub height. A relatively large discrepancy exists between the values of *St* reported by different numerical and wind-tunnel studies, ranging between 0.12 and 0.85 (e.g., Medici and Alfredsson 2006, 2008; Chamorro and Porté-Agel 2010; España et al. 2012; Iungo et al. 2013a; Chamorro et al. 2013a; Okulov et al. 2014; Foti et al. 2016; Barlas et al. 2016; Coudou et al. 2017). This emphasizes the need for further study to elucidate the underlying mechanisms leading to the development of the hub vortex. It should also be mentioned that all the above studies were performed with laboratory-scale wind turbines; therefore, it is of interest to investigate if the same periodic motions can be observed in the wake of utility-scale turbines, for which the ratio of the nacelle to the rotor is smaller than that of laboratory-scale turbines. Finally, it is also important to point out that these periodic motions observed in the central part of the near-wake are different from the random oscillations of the turbine far-wakes, often referred to as wake meandering. Meandering of turbine far-wakes is mainly caused by very large turbulent structures in the incoming boundary layer, and is discussed in detail in Sect. 2.2.2.

#### Mean Flow Distribution

Based on the conservation of angular momentum, the near-wake rotates in the opposite direction from that of the turbine blades (Manwell et al. 2010), and the amount of the rotation decreases with increasing downstream distance (Zhang et al. 2012). A speed-up region is also observed in the central part of the near-wake, particularly at higher tip-speed ratios (Krogstad and Adaramola 2012; Bastankhah and Porté-Agel 2017c). In spite of this complex nature, for the sake of simplicity, the near-wake has been modelled in some studies (e.g., Vermeulen 1980; Bastankhah and Porté-Agel 2016) with a uniform velocity-deficit distribution in the central part, and a varying velocity deficit in the side shear layers, as shown in Fig. 2 (dashed lines). Based on this simplified description, the side shear layers expand downstream until the central region with the uniform velocity deficit ultimately vanishes. Further downstream, the far-wake region, characterized by a self-similar Gaussian velocity-deficit distribution, is found. The length of the near-wake is influenced by a range of parameters such as the turbulence intensity of the incoming flow (Wu and Porté-Agel 2012), the mechanical shear generated by the turbine (Vermeulen 1980), and the turbine tip-speed ratio (Sørensen et al. 2015).

Different models have been proposed in the literature (e.g., Vermeulen 1980; Sørensen et al. 2015) to predict the length of the turbine near-wake. Based on the model proposed by Sørensen et al. (2015), the normalized near-wake length $x_n/d$ is given by

2 $$\begin{aligned} \frac{x_n}{d}=-\frac{1}{2}\left[ \left( \frac{16{\bar{u}}_c^3}{N_b\lambda C_T}\right) \ln (0.3I) + 5.5 \ln (I)\right] , \end{aligned}$$

where ${\bar{u}}_c$ is the mean convective velocity of the tip vortices normalized by the incoming flow speed (typically within the range of 0.73–0.78), $N_b$ is the number of blades, $\lambda$ is the tip-speed ratio, $C_T$ is the thrust coefficient, and *I* is the incoming streamwise turbulence intensity.

### Far-Wake

#### Mean Flow Distribution

- Velocity Distribution

In contrast to the near-wake region, the far-wake region has more universal characteristics as it is less influenced by the detailed features of the rotor (Crespo et al. 1999b; Vermeer et al. 2003). Given the fact that turbine spacing in wind farms usually falls within the range of 3 to 10 rotor diameters, wind turbines commonly operate in the far-wake of upwind turbines. As a result, understanding turbine far-wakes is essential for improving the prediction and optimization of wind-turbine power output in wind farms. In recent years, a great deal of attention has been paid to studying mean flow distribution in turbine far-wakes by means of field measurements (e.g., Barthelmie et al. 2004, 2006; Käsler et al. 2010; Trujillo et al. 2011; Hirth et al. 2012; Iungo et al. 2013b; McKay et al. 2013; Smalikho et al. 2013; Aitken et al. 2014b; Banta et al. 2015; Marathe et al. 2016; Fuertes et al. 2018), laboratory experiments (e.g., Medici and Alfredsson 2006; Chamorro and Porté-Agel 2009; Maeda et al. 2011; Chamorro et al. 2012; Aubrun et al. 2013; Singh et al. 2014; Chu and Chiang 2014; Muller et al. 2015; Li et al. 2016; Bastankhah and Porté-Agel 2017a, b, c), and numerical simulations (e.g., Jiménez et al. 2010; Wu and Porté-Agel 2011, 2012; Churchfield et al. 2012b; Lee et al. 2013; Mo et al. 2013; Castellani and Vignaroli 2013; Chatelain et al. 2013; Bastine et al. 2015; Abkar and Porté-Agel 2015a; Foti et al. 2016; Englberger and Dörnbrack 2017).

Due to the entrainment of the outer flow, the wake is found to grow in both lateral and vertical directions as it moves downstream, and the value of the streamwise velocity component increases until the wake completely recovers far downstream (Barthelmie et al. 2003; Iungo et al. 2013b; Aitken and Lundquist 2014). Early studies (e.g., Medici and Alfredsson 2006) of wind-turbine wakes in uniform inflows showed that the streamwise velocity profiles have an axisymmetric Gaussian distribution in this region. In the case of boundary-layer flows, although later studies (e.g., Chamorro and Porté-Agel 2009) showed that wake velocity profiles lose the Gaussian shape due to the incoming shear and the presence of the ground (see the schematic in Fig. 2), profiles of the velocity deficit $\varDelta {\bar{u}}$ (i.e., difference between the incoming flow speed and that of the wake) still retain the Gaussian distribution, except at the edge of the wake. The slight disagreement between the velocity-deficit profiles and the Gaussian distribution seen at the wake edges has also been reported for other types of wake flows (Pope 2000; Johansson et al. 2003; Okulov et al. 2015). One of the inherent characteristics of Gaussian profiles is self similarity, implying that the profile of velocity deficit (normalized by its maximum value) as a function of the distance from the wake centre (normalized by the wake width $\sigma$) is constant with streamwise position (Tennekes and Lumley 1972; Pope 2000). Far-wake self-similarity facilitates the development of simple analytical models for the prediction of the mean flow distribution in this region, see Sect. 2.2.3.

**Fig. 4:**
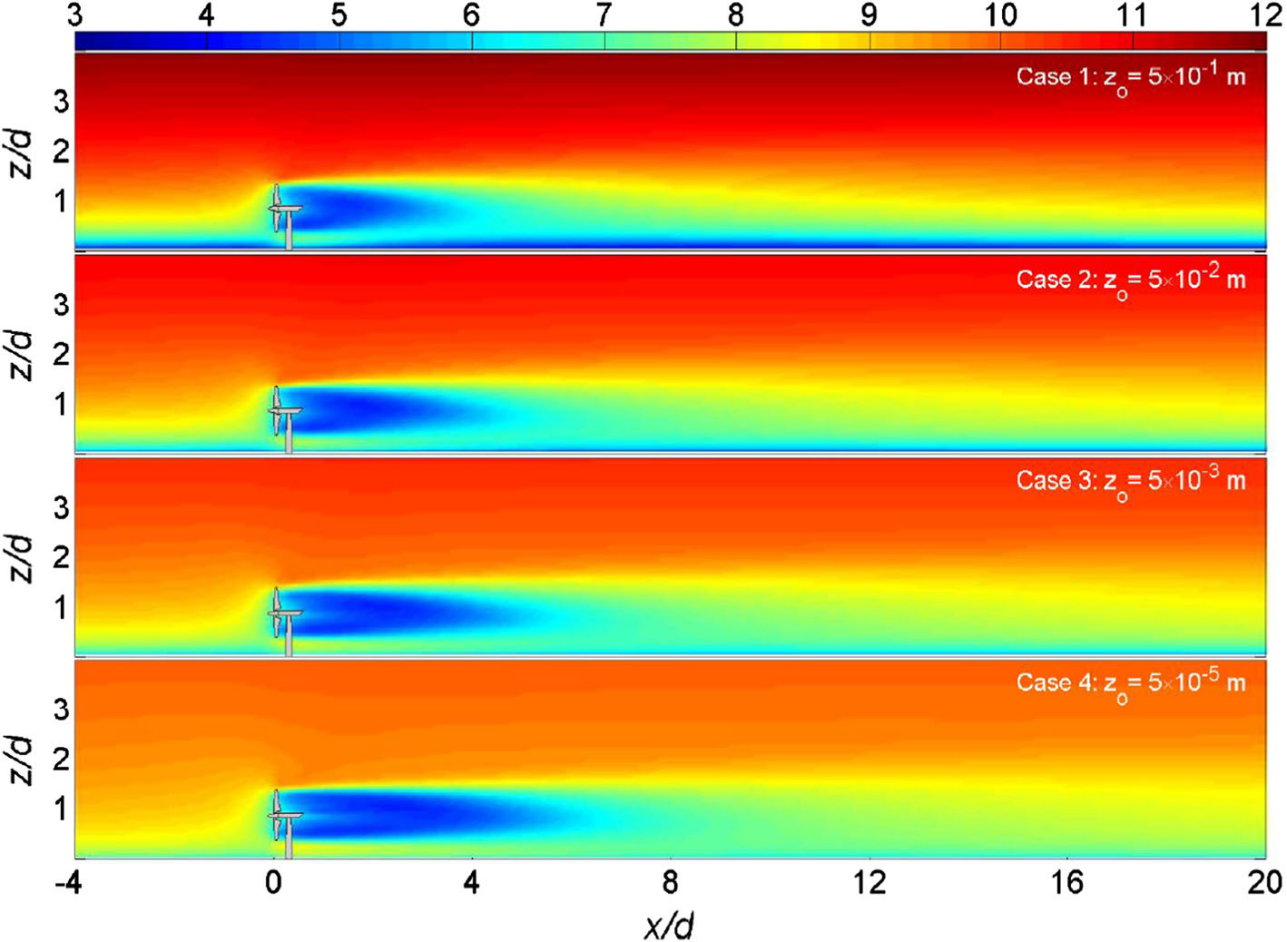
Contours of the time-averaged streamwise velocity component (in m $\hbox {s}^{-1}$) in the vertical plane normal to the rotor plane, at zero span, for different roughness lengths Figure taken from Wu and Porté-Agel (2012), in accordance with the Creative Commons Attribution (CC BY) license)

Classical theoretical studies on three-dimensional wakes of bluff bodies (e.g., disks) have shown that the wake velocity deficit $\varDelta {\bar{u}}$ decays with $x^{-2/3}$ along the rotor axis while the increase of the wake width $\sigma$ with the streamwise distance is proportional to $x^{1/3}$. These theoretical analyses are based on the assumption that shear-generated turbulence due to the wake is mainly responsible for the wake recovery, and the effect of the incoming flow turbulence is negligible. This theoretical result is confirmed by experimental studies of turbine far-wakes under laminar inflow conditions (e.g., Okulov et al. 2015). In more realistic situations when the ambient turbulence is present, however, wake recovery deviates considerably from the aforementioned theory (Wu and Faeth 1994; Bagchi and Balachandar 2004; Johnson et al. 2014). Several LES, wind-tunnel, and field studies of turbine wakes have shown that the wake width increases approximately linearly with *x*, and its recovery rate, denoted by *k*, is larger for boundary layers with higher turbulence intensity (e.g. Bastankhah and Porté-Agel 2014b; Fuertes et al. 2018). This is the main reason why turbine wakes in a rough boundary layer recover more rapidly than those in a smooth boundary layer (Chamorro and Porté-Agel 2009; Wu and Porté-Agel 2012; Barlas et al. 2016). This is illustrated in Fig. 4, showing contours of the time-averaged streamwise velocity component for the wake of a wind turbine installed over flat terrain with different roughness lengths. This effect explains why, in general, the capacity density of offshore wind farms is smaller than that of their onshore counterparts.

b. Turbulence Distribution

**Fig. 5:**
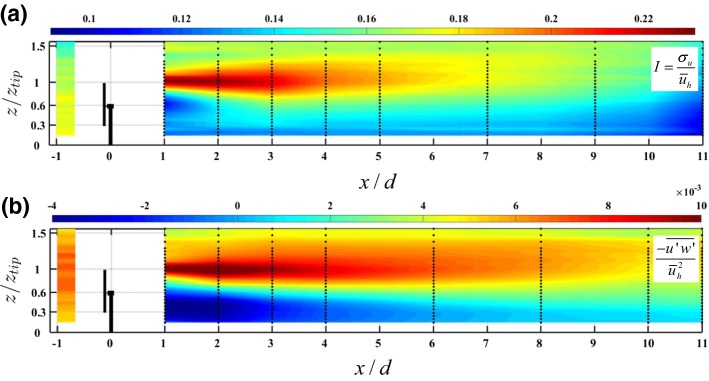
Distribution of, **a** streamwise turbulence intensity $I=\sigma _u/{\bar{u}}_h$, and **b** normalized kinematic vertical turbulent momentum flux $\overline{u'w'}/{\bar{u}}^2_h$, in a vertical plane at zero span Figure reprinted from Barlas et al. (2016) with the permission of Springer Nature

In addition to the far-wake mean velocity distribution, turbulence characteristics of far-wakes have been extensively studied in the literature. Specifically, the following turbulence quantities are mostly considered:

- *Streamwise turbulence intensity* (i.e., $I=\sigma _u/{\bar{u}}_h$): wind turbine far-wakes are known to have a high turbulence intensity with respect to the incoming flow, in particular the upper part of the wake. The increased turbulence intensity in far-wakes has received considerable attention in the literature as it can induce harmful unsteady loads on downwind turbines. The turbulence intensity added by the turbine $\varDelta I$ is given by (Frandsen 2007),
  3 $$\begin{aligned} \varDelta I=\sqrt{I_w^2-I^2}, \end{aligned}$$
  where $I_w$ is the streamwise turbulence intensity in the wake. Under uniform inflow conditions, $I_w$ has a double Gaussian profile with the maximum values occurring at the edge of the wakes (Maeda et al. 2011; Li et al. 2016). In boundary-layer flows, while the maximum value of the turbulence intensity usually occurs close to the upper edge of the wake as shown in Fig. 5a, the turbulence is suppressed by the turbine in regions close to the ground. The value of $\varDelta I$ reaches its maximum in the range of two to four rotor diameters downstream at the top-tip level, coinciding with the transition between the near-wake and the far-wake. The peak of *I* therefore occurs earlier for incoming boundary layers with higher turbulence intensity since the near-wake is shorter in this case (Wu and Porté-Agel 2012). Further downstream, the value of turbulence intensity monotonically decreases with *x* in the far-wake. Different empirical and semi-empirical models have been proposed in the literature to predict the variation of $\varDelta I$ with *x* in turbine far-wakes, see Quarton (1989), Hassan (1993), Crespo and Hernández (1996), Xie and Archer (2015) and Qian and Ishihara (2018), among others, for more information on this topic.
- *Turbulent momentum flux* (i.e., $\rho \overline{u'v'}$ in the lateral direction and $\rho \overline{u'w'}$ in the vertical one, where primes indicate turbulent fluctuations): the spatial distribution of the turbulent momentum flux in turbine wakes reflects the entrainment of air from the outer flow towards the wake centre. Akin to the streamwise turbulence intensity, the magnitude of the momentum flux is greater at the edges of the far-wake, especially close to the upper edge of the wake where the wind shear is greater, as seen in Fig. 5b.
- *Turbulence kinetic energy (TKE)* (i.e., $e=\frac{1}{2}(\overline{u'^2}+\overline{v'^2}+\overline{w'^2})$): the analysis of the TKE budget provides insights into the production and transportation of turbulence structures in wind-turbine wakes. Prior studies (e.g., Wu and Porté-Agel 2012; Kang et al. 2014; Xie and Archer 2015) showed that the TKE production has high values in the near-wake, particularly in the upper edge of the wake, where mean shear and turbulent fluxes are significant. The generated TKE at the edge of the turbine wake is then advected by the mean wind downstream.

#### Wake Meandering

**Fig. 6:**
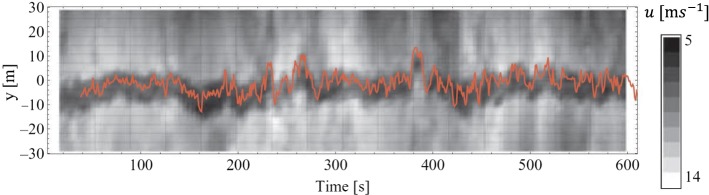
Wake temporal oscillations at three rotor diameters downwind of a turbine. Velocity contours obtained from lidar measurements in the field are shown in greyscale, and the red line indicates the temporal variation of the wake centre predicted by the DWM model Figure reprinted from Bingöl et al. (2010) with the permission of John Wiley and Sons, Inc

Wake meandering relates to the random unsteady oscillations of the entire wake with respect to the time-averaged wake centreline. These random oscillations lead to strong turbulence generation and consequently harmful unsteady loads on downwind turbines (Ainslie 1988; Larsen et al. 2008; Churchfield et al. 2012b). There is almost unanimous agreement in the wind energy community that wake meandering is caused by very large turbulent eddies in the incoming boundary layer. Ainslie (1988) is perhaps the first study to incorporate the effect of wake meandering into the wake-flow prediction. Later, Larsen et al. (2008) postulated that, while the wake recovery is governed by small turbulent eddies, the whole wake is advected passively by turbulent eddies larger than twice the rotor diameter. Therefore, if the low frequency variation of the incoming flow is known, one can model random oscillations of the turbine wake as a passive scalar. This study became the basis of the dynamic wake meandering (DWM) model that was later validated and used to predict instantaneous wake-centre position (Trujillo et al. 2011; Keck et al. 2014, 2015) and unsteady loads on downwind turbines (Larsen et al. 2013) in field. Instead of the incoming flow speed, Bingöl et al. (2010) estimated the wake transportation based on the wake model of Jensen (1983). Although this assumption is not consistent with the passive scalar hypothesis, they reported a better agreement between DMW predictions and field measurements. The DMW predictions in comparison with field measurements are shown in Fig. 6.

The connection between the incoming flow characteristics and wake meandering has been further studied in a series of recent wind-tunnel studies. España et al. (2011) experimentally confirmed that wake meandering does not occur unless turbulent eddies much larger than the turbine rotor diameter exist in the incoming flow. Muller et al. (2015) showed a spectral coherency at large wavelengths between the incoming boundary-layer flow and the instantaneous wake-centre position. España et al. (2012) and Bastankhah and Porté-Agel (2017c) investigated the amplitude of wake meandering under different conditions, and showed that the amplitude of the wake meandering increases as the wake moves downstream. Moreover, even though the wake-meandering amplitude is sensitive to the incoming flow conditions (España et al. 2012), it does not depend on turbine operating conditions (e.g., tip-speed ratio, yaw angle) (Bastankhah and Porté-Agel 2017c).

One of the commonly reported characteristics of wake meandering is that lateral displacements are much more pronounced than vertical ones. España et al. (2012) argued that this difference is due to the higher value of $\sigma _v$ than $\sigma _w$ in turbulent boundary-layer flows. Bastankhah and Porté-Agel (2017b) hypothesized that this difference is due to the lateral meandering tendency of very-large-scale motions (VLSMs) present in the incoming boundary layer. VSLMs or superstructures are very long low- and high-momentum structures observed both in the atmospheric surface layer and the logarithmic region of a laboratory-scale boundary layer (Hutchins and Marusic 2007; Hutchins et al. 2012). The length scale of VLSMs can exceed $20\delta$, where $\delta$ is the boundary-layer thickness (Fang and Porté-Agel 2015), and they are very energetic structures since they account for a considerable share of the TKE and shear stress (Kim and Adrian 1999; Guala et al. 2006; Lee and Sung 2011). The interaction of VLSMs with wind-turbine wakes might explain another feature of turbine-wake meandering: namely, the fact that the mean wake cross-section is not stretched laterally in spite of large meandering motions in the lateral direction.

#### Analytical Wake Modelling

As discussed in Sect. 1, some applications such as wind-farm-layout optimization require the prediction of wake flows for many (on the order of thousands or more, depending on the optimization technique) scenarios including, but not limited to, multiple layouts and variations in wind direction, wind speed, and thermal stratification. Such optimization can only be achieved using simple and computationally inexpensive wake models. These models can be divided into two main categories: (i) empirical models, and (ii) analytical models.

Empirical models have been used (e.g., Baker and Walker 1984; Högström et al. 1988; Magnusson and Smedman 1999; Barthelmie et al. 2003; Zhang et al. 2013b; Iungo and Porté-Agel 2014; Aitken et al. 2014b) to mainly estimate the variation of the wake-centre velocity deficit with the streamwise distance from the turbine rotor. Based on these models, the velocity deficit is generally assumed to have a power-law relationship with *x*, which is written as

4 $$\begin{aligned} \frac{\varDelta {\bar{u}}}{{\bar{u}}_{\infty }}=A\left( \frac{x}{d}\right) ^n, \end{aligned}$$

where *A* and *n* are coefficients obtained from experimental and numerical data.

Unlike empirical models, whose model equation is obtained solely by fitting experimental or numerical data, analytical wake models are derived based on flow governing equations and, therefore, have a superior ability to capture the physics. The wind-energy literature is enriched with many studies aimed at developing analytical models for wind-turbine wakes, see Vermeulen (1980), Jensen (1983), Katić et al. (1986), Ainslie (1988), Larsen (1988), Frandsen et al. (2006), Ott (2011), Bastankhah and Porté-Agel (2014b), and Tian et al. (2015). For the sake of brevity, here, we review those that attracted the most attention: Jensen (1983), Frandsen et al. (2006) and Bastankhah and Porté-Agel (2014b). More information on analytical wake models can be found in, e.g., Crespo et al. (1999b), Barthelmie et al. (2003), and Göçmen et al. (2016).

Jensen (1983) developed a pioneering turbine-wake model, which has been extensively used in the literature and commercial software (e.g., WAsP, WindPRO, WindSim, WindFarmer, and OpenWind). The Jensen model is obtained by applying the conservation of mass to a control volume downwind of the wind turbine, and then using the so-called Betz theory to relate the wind speed just behind the rotor to the turbine thrust coefficient $C_T$ (Katić et al. 1986). It also assumes a top-hat distribution for the velocity deficit in the wake for the sake of simplicity. The normalized velocity deficit based on this model is given by

5 $$\begin{aligned} \frac{\varDelta {\bar{u}}}{{\bar{u}}_{\infty }}=\frac{1-\sqrt{1-C_T}}{\left( 1+2k_tx/d\right) ^2}, \end{aligned}$$

where $C_T$ is the thrust coefficient of the turbine, ${\bar{u}}_{\infty }$ is the mean incoming flow speed, and $\varDelta {\bar{u}}={\bar{u}}_{\infty }-{\bar{u}}$. The wake width is assumed to grow linearly with downwind distance and, therefore, the wake growth rate, $k_t$, is constant. Jensen (1983) suggested that $k_t=0.1$, whereas values of 0.04 or 0.05 for $k_t$ in offshore cases and 0.075 for onshore cases are suggested in the later literature (Barthelmie et al. 2009; Göçmen et al. 2016). Alternatively, $k_t$ can be estimated by the ratio of the friction velocity to the streamwise velocity component at the hub height for the incoming boundary layer (Frandsen 1992). For a logarithmic wind profile, this approximately gives

6 $$\begin{aligned} k_t\approx \frac{0.5}{\ln (z_h/z_0)}, \end{aligned}$$

where $z_h$ and $z_0$ are the turbine hub height and the roughness length, respectively. Peña and Rathmann (2014) extended the above relationship to account for the effect of thermal stratifications on the wake growth rate.

Frandsen et al. (2006) used the conservation of mass and momentum for a control volume around the turbine, with the same top-hat shape assumed for velocity-deficit profiles in the wake. Based on this work, the normalized velocity deficit is given by

7 $$\begin{aligned} \frac{\varDelta {\bar{u}}}{{\bar{u}}_{\infty }}=\frac{1}{2}\left( 1-\sqrt{1-\frac{2 C_T}{\beta +\alpha x/d}}\right) , \end{aligned}$$

where $\alpha$ is of order of $10k_t$ and

8 $$\begin{aligned} \beta =\frac{1+\sqrt{1-C_T}}{2\sqrt{1-C_T}}. \end{aligned}$$

Note that $\beta$ is meaningful only for values of $C_T$ smaller than one.

As a result of the assumption of a top-hat distribution for wake velocity-deficit profiles, these models tend to underestimate the velocity deficit at the wake centre and overestimate it at the edges of the wake. Moreover, Bastankhah and Porté-Agel (2014b) showed that top-hat models make the power predictions of downwind turbines unrealistically sensitive to the lateral position of turbines with respect to each other. Different numerical and experimental data were used by Bastankhah and Porté-Agel (2014b) to show that self-similar Gaussian distribution can acceptably represent velocity-deficit profiles in turbine far-wakes. The normalized velocity deficit is therefore given by

9 $$\begin{aligned} \frac{\varDelta {\bar{u}}}{{\bar{u}}_{\infty }}=C(x)\,\text {exp}\left( {-\frac{r^2}{2\sigma ^2}}\right) , \end{aligned}$$

where $\sigma$ is the wake width. A linear wake growth rate is assumed for the wake, since this is in agreement with wind-tunnel measurements (Chamorro and Porté-Agel 2010) and LES data (Wu and Porté-Agel 2011). Hence, $\sigma$ is given by

10 $$\begin{aligned} \frac{\sigma }{d}=k\frac{x}{d}+\epsilon , \end{aligned}$$

where *k* is the wake growth rate, and $\epsilon$ is the initial wake width, equal to $0.2\sqrt{\beta }$. The conservation of mass and momentum in an integral form is expressed by

11 $$\begin{aligned} T=\frac{\uppi d^2}{8}\rho C_T {\bar{u}}^2_{\infty }=\rho \int {\bar{u}}\left( {\bar{u}}_{\infty }-u\right) \text {d}A, \end{aligned}$$

where *T* is the turbine thrust force. Inserting Eq. 9 into Eq. 11 yields

12 $$\begin{aligned} \frac{\varDelta {\bar{u}}}{u_{\infty }}=\left( 1-\sqrt{1-\frac{C_T}{8(\sigma /d)^2}}\right) \text {exp}\left( -\left\{ \frac{y^2}{2\sigma ^2} + \frac{(z-z_h)^2}{2\sigma ^2} \right\} \right) , \end{aligned}$$

where $\sigma$ is given by Eq. 10.

In order to use this model to predict the wake velocity distribution, the value of the wake growth rate *k* has to be estimated for each case. Note that the original version of the model expressed by Eq. 12 assumes that the wake growth rate *k* is the same in both lateral and vertical directions. Abkar and Porté-Agel (2015a) and Xie and Archer (2015) showed, however, that the wake width in the vertical direction can be different from that in the lateral direction due to the effect of the ground or thermal stratification. Hence, for the sake of generality, the model can be written as

13 $$\begin{aligned} \frac{\varDelta {\bar{u}}}{u_\infty }=\left( 1-\sqrt{1-\frac{C_T}{8 \left( \sigma _y\sigma _z/d^2\right) }}\right) \text {exp}\left( -0.5\left[ \left( \frac{y}{\sigma _y}\right) ^2+\left( \frac{z-z_h}{\sigma _z}\right) ^2\right] \right) , \end{aligned}$$

where $\sigma _y$ and $\sigma _z$ are given by

14a $$\begin{aligned} \frac{\sigma _y}{d}&= k_y\frac{x}{d}+\epsilon , \end{aligned}$$

14b $$\begin{aligned} \frac{\sigma _z}{d}&= k_z\frac{x}{d}+\epsilon . \end{aligned}$$

Here, $k_y$ and $k_z$ are wake growth rates in the *y* and *z* directions, respectively, and as mentioned earlier, $\epsilon =0.2\sqrt{\beta }$.

**Fig. 7:**
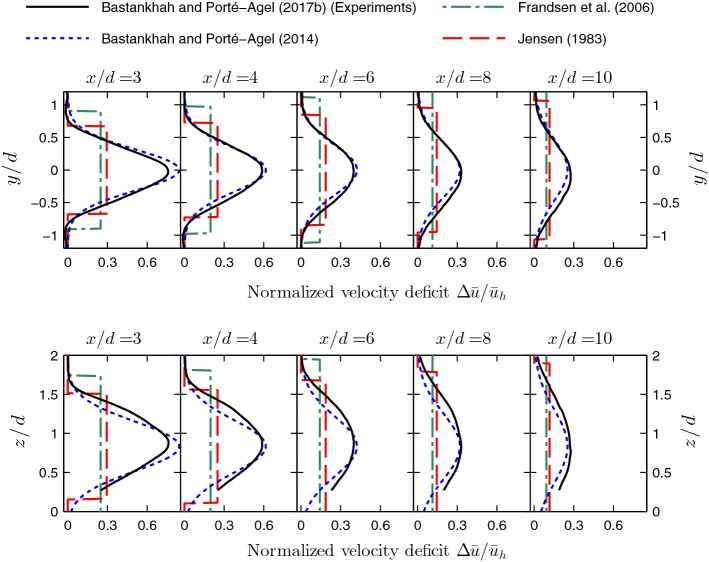
Lateral (top) and vertical (bottom) profiles of the normalized velocity deficit through the hub level at different downwind locations. The data obtained from wind-tunnel measurements (Bastankhah and Porté-Agel 2017b) are shown by black solid lines. The predictions of the analytical models developed by Jensen (1983), Frandsen et al. (2006) and Bastankhah and Porté-Agel (2014b) are shown by red dashed lines, green dash-dot lines and blue dashed lines, respectively

Figure 7 shows the predictions of the analytical models reviewed above in comparison with the wind-tunnel data recently reported by Bastankhah and Porté-Agel (2017b). The model inputs are determined based on the incoming boundary-layer flow conditions as well as turbine operating conditions reported in the mentioned study. The growth rate of the top-hat wake $k_t$ is calculated according to Eq. 6, while the wake growth rate *k* for the last model with a Gaussian velocity deficit profile is estimated to be 0.022 based on the wind-tunnel data.

A key parameter of this empirical model is the wake growth rate *k*, which depends on the turbulence intensity in the incoming flow. Niayifar and Porté-Agel (2016) used LES data to propose the following empirical linear relation to estimate *k* as a function of the streamwise turbulence intensity *I* (for $0.06<I<0.15$),

15 $$\begin{aligned} k\approx \alpha _1 I+\alpha _2, \end{aligned}$$

with $\alpha _1=0.38$ and $\alpha _2=0.004$. A recent field study of wind-turbine wakes using two nacelle-mounted lidars (Fuertes et al. 2018) has reported a reasonable fit of the measurements using Eq. 15 for the growth rate (with $\alpha _1=0.35$ and $\alpha _2=0$).

It should be mentioned that, even though the streamwise turbulence intensity is extensively used in analytical modelling of wind-turbine wakes (as discussed in Sects. 2, 3), some studies (e.g., Larsen et al. 2008) have suggested that the spanwise and vertical velocity component fluctuations play a dominant role on the structure and dynamics of wind-turbine wakes. Considering this, Cheng and Porté-Agel (2018) proposed a physics-based analytical model for the wake expansion based on Taylor’s diffusion theory (Taylor 1922).

#### Yawed Conditions

Power losses due to complex interactions of wind-turbine wakes in wind farms call for the development of new effective wake mitigation strategies. A promising approach for achieving this goal is to intentionally hinder the performance of single wind turbines in order to improve the whole wind-farm power production. Based on this approach, different techniques have been described in the literature, such as the active control of the blade pitch, tilt angle or yaw angle of wind turbines. In particular, yaw angle control is nowadays considered as an effective strategy for deflecting the wakes away from downwind turbines (Dahlberg and Medici 2003; Adaramola and Krogstad 2011; Ozbay et al. 2012; Schottler et al. 2017). Several numerical and experimental studies have recently shown that overall power production in wind farms can be considerably improved through yaw angle control (e.g., Campagnolo et al. 2016; Park and Law 2016a, b; Fleming et al. 2018; Bastankhah and Porté-Agel 2019). Although more study is required to address concerns about turbine structural loads under yaw misalignment, Kragh and Hansen (2014), Gebraad et al. (2014), Bastankhah and Porté-Agel (2014a), Fleming et al. (2014), amongst others, have shown that, under certain circumstances, yawing turbines may even lead to the reduction of loads.

The literature on the far-wake of yawed turbines is reviewed below, while regarding the performance of yawed rotors and their near-wakes, the reader is referred to Burton et al. (1995), Grant and Parkin (2000), Haans et al. (2005), Haans et al. (2006), Maeda et al. (2008), Krogstad and Adaramola (2012), McWilliam et al. (2011), Micallef et al. (2013), Campo et al. (2015), Branlard and Gaunaa (2016), Bastankhah and Porté-Agel (2017c), and Felli and Falchi (2018), amongst others.

**Fig. 8:**
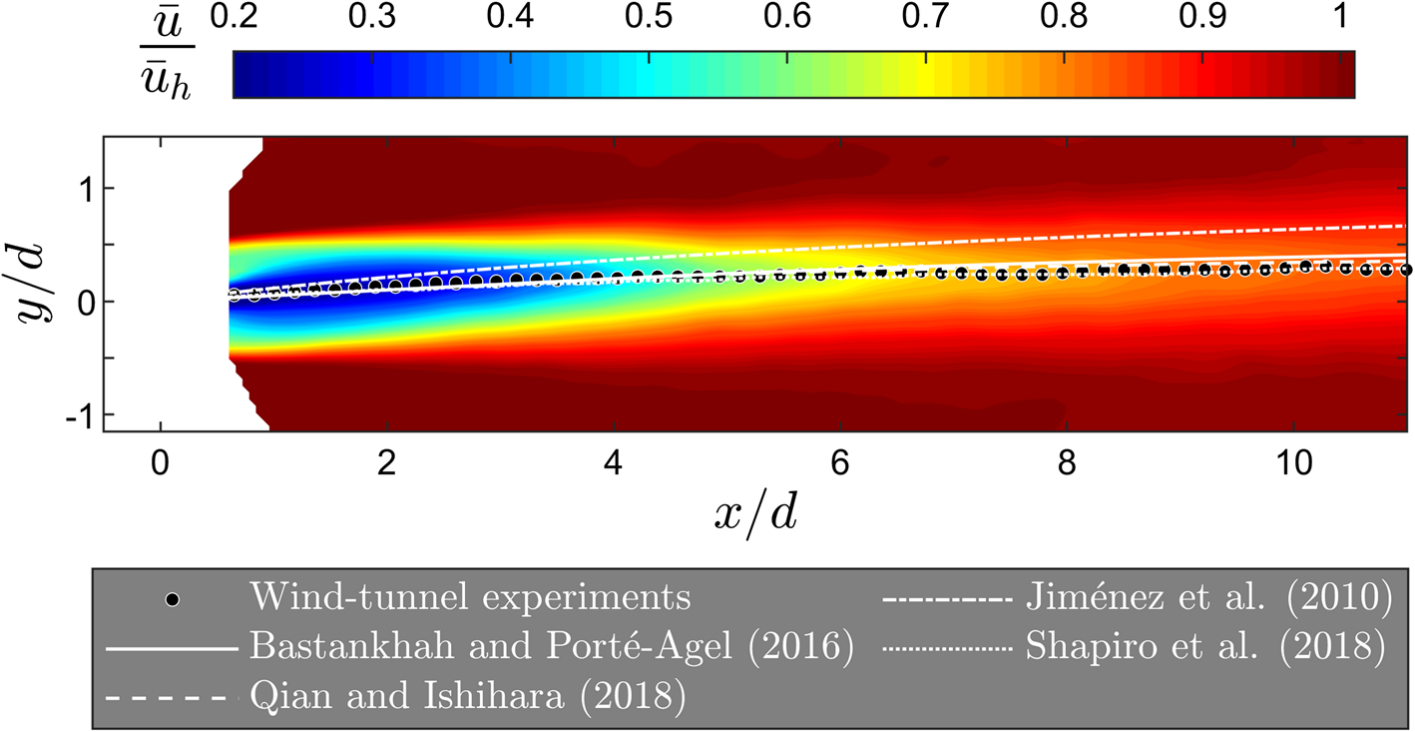
Contours of the normalized streamwise velocity component on a horizontal plane at hub height for a yawed turbine with $\gamma =20^{\circ }$ obtained with wind-tunnel measurements. The wake centre trajectories based on wind-tunnel experiments as well as different analytical wake models are also shown

Far-wake flow distribution and the wake deflection under yawed conditions have been the subject of several studies such as Medici and Alfredsson (2006), Fleming et al. (2014), and Marathe et al. (2016). These studies have shown that wake deflection increases as the wake moves downstream. This can be seen in Fig. 8, which shows contours of the normalized streamwise velocity component on a horizontal plane at hub height for a turbine with a yaw angle of $20^{\circ }$, obtained with wind-tunnel experiments reported by Bastankhah and Porté-Agel (2016). In general, the amount of the wake deflection has been found to increase with: (i) the increase of yaw angle (Jiménez et al. 2010; Fleming et al. 2014), (ii) the increase of thrust coefficient (Jiménez et al. 2010), (iii) the decrease in incoming turbulence intensity (Bastankhah and Porté-Agel 2016), and (iv) the increase of thermal stability (Churchfield et al. 2016; Vollmer et al. 2016). This suggests that the yaw-angle control of wind turbines is more plausible for offshore wind farms, or for turbines operating in a stable boundary layer. Another feature of yawed turbine wakes is that the maximum value of the wake skew angle does not occur at the wake centre where the velocity deficit is maximum, but occurs in one side of the wake where the second derivative of the lateral profiles of the streamwise velocity component is zero (Bastankhah and Porté-Agel 2016).

While turbine wakes are slightly affected by small yaw angles (e.g., less than $10^\circ$), they undergo fundamental changes under highly yawed conditions (e.g., greater than $20^\circ$). For instance, the wake cross-section of a highly-yawed turbine has a kidney shape due to the presence of a counter-rotating vortex pair (Howland et al. 2016; Bastankhah and Porté-Agel 2016; Churchfield et al. 2016; Wang et al. 2017). Bastankhah and Porté-Agel (2016) showed that the formation of counter-rotating vortex pairs needed to satisfy continuity in any free shear flow with a strong variation in the cross-wind velocity component such as turbine wakes under highly yawed conditions and cross-flow jets. They also employed wind-tunnel data and a theoretical analysis based on the potential theory to show that, in addition to lateral displacements, turbine wakes have vertical displacements under highly yawed conditions. The yaw-angle direction affects both the magnitude and direction of horizontal (Fleming et al. 2014; Gebraad et al. 2014; Schottler et al. 2017) and vertical (Bastankhah and Porté-Agel 2016) wake displacements.

The lateral wake deflection of turbines wakes under yawed conditions can be simply explained by the conservation of momentum. This raises the possibility of deriving simple analytical models to predict the magnitude of the far-wake deflection for yawed turbines. In the pioneering work of Jiménez et al. (2010), a simple relationship for the wake skew angle is suggested based on conservation of mass and momentum for the wake of a yawed turbine with top-hat velocity profiles. Gebraad et al. (2014) combined the findings of the above-mentioned work with the model developed by Jensen (1983) to estimate the wake of a yawed turbine. Bastankhah and Porté-Agel (2016) later used the budget study of RANS equations to develop an analytical model that predicts the wake-flow distribution in yawed conditions. While wake self-similar characteristics (Gaussian distributions for velocity deficit and skew angle profiles) were used to model flow distribution in the far-wake region, vortex-theory predictions based on Coleman et al. (1945) were used to estimate the near-wake skew angle. Recently, Shapiro et al. (2018) developed a model that implements a different approach to predict the near-wake skew angle. They treated a yawed turbine as a lifting line, with an elliptic distribution of force, and used Prandtl’s lifting line theory to determine the near-wake skew angle. Qian and Ishihara (2018) have more recently proposed a wake model for yawed turbines by assuming a Gaussian distribution for velocity-deficit profiles and a top-hat shape for those of the wake skew angle. Wake-centre trajectories predicted by the above-mentioned models are compared in Fig. 8 with wind-tunnel experiments reported in Bastankhah and Porté-Agel (2016).

**Fig. 9:**
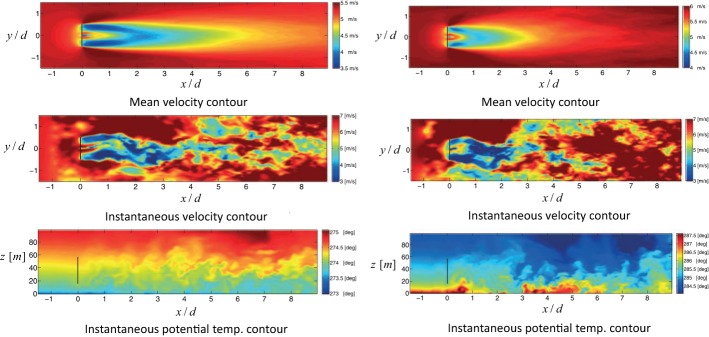
Contour plots of the 10-min average wake velocity (top) and instantaneous velocity (middle) on a horizontal plane at hub height, as well as the instantaneous potential temperature on a vertical plane at zero span (bottom). Results are from large-eddy simulations of wake flow under very stable (left) and unstable (right) atmospheric conditions Figure reprinted from Machefaux et al. (2016) with the permission of John Wiley and Sons, Inc

### Thermal Effects

Thermal stability of the ABL is known to play a significant role on wind-turbine performance as well as the structure and dynamics of wind-turbine wake flows. The main effects on stand-alone turbines are due to the changes in mean shear and turbulence intensity of the incoming flow, associated with changes in thermal stability, as shown in numerous wind-tunnel experiments (Chamorro and Porté-Agel 2010; Zhang et al. 2013b; Hancock and Pascheke 2014), field observations (Baker and Walker 1984; Magnusson and Smedman 1999; Iungo and Porté-Agel 2014; Aitken et al. 2014b; Machefaux et al. 2016), and numerical simulations (Churchfield et al. 2012b; Keck et al. 2014; Aitken et al. 2014a; Mirocha et al. 2015; Abkar and Porté-Agel 2015a; Machefaux et al. 2016).

Wind-turbine wakes recover considerably faster and display stronger meandering in the convective boundary layer (CBL), compared with the neutral ABL and the stable boundary layer (SBL), as shown in several studies (e.g., Baker and Walker 1984; Ainslie 1988; Magnusson and Smedman 1994; Hancock and Pascheke 2014; Keck et al. 2014; Aitken et al. 2014a; Abkar and Porté-Agel 2015a; Machefaux et al. 2016). Figure 9 clearly illustrates the effect of thermal stability on the recovery of a wind-turbine wake simulated using LES. This trend is mainly attributed to the relatively higher turbulence intensity in the CBL (Zhang et al. 2013b; Iungo and Porté-Agel 2014; Abkar and Porté-Agel 2015a), which leads to enhanced turbulent mixing, flow entrainment, wake meandering and wake recovery, compared to the neutral ABL and SBL (Baker and Walker 1984; Ainslie 1988; Zhang et al. 2013b; Iungo and Porté-Agel 2014; Hancock and Pascheke 2014; Abkar and Porté-Agel 2015a; Machefaux et al. 2016). It should be noted that, as discussed in Sect. 2.2.3 and in agreement with the dynamic wake-meandering model of Larsen et al. (2008) and the analytical model of Cheng and Porté-Agel (2018), the radial (spanwise and vertical) turbulence intensity is expected to play a more important role than the streamwise turbulence intensity on both wake meandering and wake recovery.

Some efforts towards mathematical modelling of the aforementioned thermal effects have been made. Ainslie (1988) developed a wake model in which the length and velocity scales used to define the “eddy diffusivity of momentum” are a function of the thermal stability. Based on this work, Keck et al. (2014) modified the dynamic wake-meandering model to take into account the effect of atmospheric stability on wake meandering and wake recovery. They validated the results of their model (for wake meandering, as well as velocity and turbulence intensity profiles) against LES and field data. Abkar and Porté-Agel (2015a) modified the analytical wake model of Bastankhah and Porté-Agel (2014b) in such a way that it considers different wake recovery rates (and wake widths) in the lateral and vertical directions. Consequently, the velocity-deficit distribution (in the normal-to-streamwise plane) is considered to have a 2D elliptical Gaussian shape instead of an axisymmetric one.

Atmospheric stability also has an influence on the fatigue loading of wind-turbine blades, as discussed in Lavely et al. (2011), Churchfield et al. (2012b), Sathe et al. (2013), and Lee et al. (2013). For example, increased atmospheric stability has been shown to increase the fatigue loading associated with mean wind shear, while it decreases the fatigue associated with turbulence (Sathe et al. 2013). It should be mentioned that the SBL is characterized by both a large vertical shear, associated with the relatively shallow depth, as well as a large lateral shear, owing to the change of wind direction with height produced by the Coriolis force. Abkar et al. (2018) have recently modified the analytical wake model of Bastankhah and Porté-Agel (2014b) to account for the wake deformation induced by Coriolis effects. In some cases, the SBL can be so shallow that part of the rotor disk (or even the entire rotor, as shown in some field data) lies above it; in such cases, although the flow above the SBL is non-turbulent, the intermittent turbulence and bursting events commonly found at the top of the SBL can pose hazardous structural threats to wind-turbine blades (Zhou and Chow 2012).

## Flow Inside and Around Wind Farms

### Flow Regions Inside and Around a Wind Farm

**Fig. 10:**
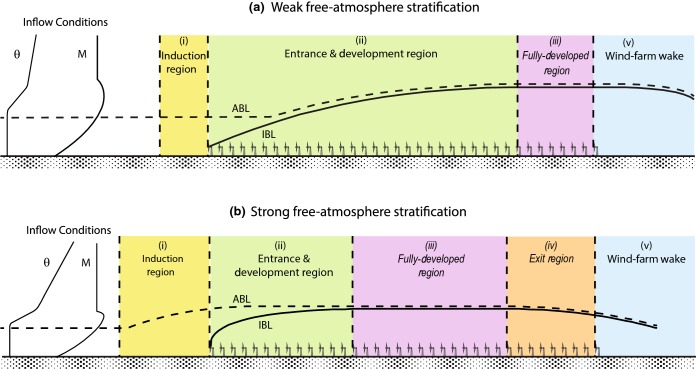
Schematic showing different flow regions caused by the interaction of a very large wind farm with a conventionally-neutral ABL under weak free-atmosphere stratification (**a**) and strong free-atmosphere stratification (**b**). The dashed and continuous lines represent the top of the ABL and the farm-induced IBL, respectively. Inflow conditions are represented by vertical profiles of the mean potential temperature ($\theta$) and mean wind speed (*M*). In italics: regions that might or might not be present in a wind farm, depending on the wind-farm size and the free-atmosphere stratification Figure modified from Wu and Porté-Agel (2017)

As a result of its interaction with a wind farm, the ABL undergoes important changes, which in turn modify the performance of the wind turbines with respect to that of hypothetical stand-alone wind turbines placed in the same undisturbed boundary layer. In the case of flat terrain, several distinct flow regions emerge from that interaction, as illustrated in Fig. 10. A short description of these flow regions is given below.

**Fig. 11:**
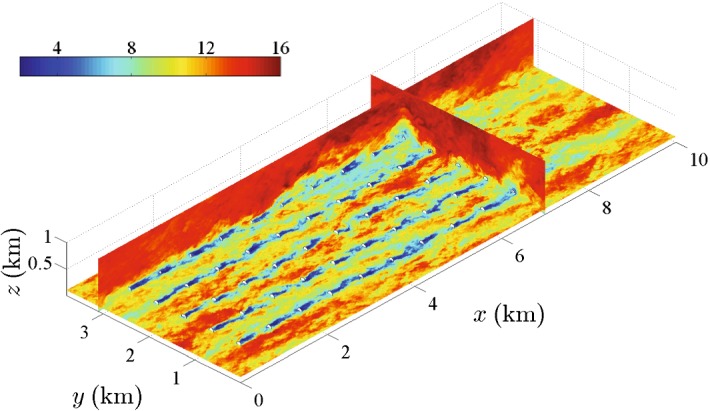
Horizontal and vertical contours of the instantaneous wind speed (in $\text {m s}^{-1}$) in the entrance region of a wind farm simulated with LES using the Smagorinsky turbulence model and a standard actuator-disk model. Downstream of the wind farm, a fringe region is used to introduce the inflow, computed in a separate precursor simulation Figure taken from Goit et al. (2016), in accordance with the Creative Commons Attribution (CC BY) license

- *The wind-farm induction zone*: immediately upwind of the wind farm, besides the blockage effect of individual wind turbines described in Sect. 2, there is a cumulated blockage effect induced by the wind farm as a whole. This produces a deceleration of the incoming boundary-layer flow and its deflection upwards and sideways due to mass conservation. The extent and strength of the farm induction region depends on the wind-farm size, layout, wind direction, turbine spacing, and thrust coefficient (Branlard 2017). Using both a cylindrical vortex wake model and actuator-disk simulations, Branlard (2017) showed that the wind speed at a distance of 2.5*d* in front of a wind farm may easily be reduced by 3% with respect to the actual incoming flow speed. This reduction is similar to that measured in the field (Bleeg et al. 2018) and simulated using the RANS approach (Bleeg et al. 2018) as well as LES (Wu and Porté-Agel 2017), for the case of wind farms inside a neutral ABL capped with a thermally-stratified free atmosphere (also known as a conventionally-neutral ABL) with relatively small lapse rates. It has also been shown that the extent and strength of the induction region, and therefore its effect on power losses in the wind farm, can be substantially larger in the case of a shallow conventionally-neutral ABL with relatively strong free-atmosphere stratification. This is due to the fact that, in such a case, the flow becomes subcritical and, therefore, the upward flow deflection induced by the wind farm triggers standing gravity waves, which are responsible for further flow blockage, also referred to as *choking* (Smith 2010; Wu and Porté-Agel 2017; Allaerts and Meyers 2018).
- *The entrance and flow development region*: downwind of the leading edge of the wind farm, the extraction of momentum by the wind turbines leads to the formation of turbine-wake flows, as described in Sect. 2. Within some downwind distance, the flow is dominated by the presence of individual turbine wakes and, therefore, it remains strongly heterogeneous in all directions (Fig. 11). Further into the wind farm, the different turbine wakes expand and interact with other wakes, leading to a flow that remains highly heterogeneous at turbine level, but becomes more homogeneous at the upper part of the flow region influenced by the turbine wakes. This region can be considered as an internal boundary layer (IBL), similar to that found downwind of smooth-to-rough surface transitions (see Garratt (1992) for a review on the IBL). The IBL grows with downwind distance from the transition, *x*, following Elliott’s $x^{4/5}$ power law (Elliott 1958), as shown in recent LES studies (e.g., Stevens 2016; Allaerts and Meyers 2017; Wu and Porté-Agel 2017). For large enough wind farms, the IBL reaches the top of the ABL and starts to grow with it by entraining momentum from the free atmosphere. This growth of the ABL continues further downwind until the flow reaches the fully-developed state described below. More details on the entrance and flow-development region are given in Sect. 3.3.
- *The fully-developed region*: in this region, the entire boundary-layer flow is fully adjusted to the wind farm and, therefore, the spanwise- and row-averaged flow is homogeneous in the streamwise direction. The power extraction by the wind turbines is balanced exclusively by the turbulent vertical transport of kinetic energy entrained from the flow above. This asymptotic case, also referred to as *infinite wind-farm case*, has been extensively studied in the literature (e.g., Frandsen 1992; Calaf et al. 2010; Lu and Porté-Agel 2011; Calaf et al. 2011; Lu and Porté-Agel 2015; Abkar and Porté-Agel 2013). Calaf et al. (2010) stated that the fully-developed regime could be attained after distances of one order of magnitude larger than the ABL height; however, recent LES studies of very large wind farms in the conventionally-neutral ABL have shown that much longer distances (about two orders of magnitude and larger) are required to achieve the fully-developed wind-farm flow (Wu and Porté-Agel 2017). Markfort et al. (2012) used scaling analysis to propose a sparse canopy model similar to that of Belcher et al. (2003) to estimate the adjustment length required for wind-farm flows to undergo transition to the fully-developed regime under neutral stratification. More details on the fully-developed wind-farm flow are given in Sect. 3.2.
- *The exit region*: this region has been observed in simulations of very large wind farms placed in the conventionally-neutral ABL capped with a strongly-stratified free atmosphere (Wu and Porté-Agel 2017). In that particular situation, the vertical deflection of the subcritical flow in the downwind region of the wind farm triggers a standing gravity wave whose effects propagate upwind. As a result, a large accelerating exit region upwind of the trailing edge is formed, leading to an improvement of the wind-turbine performance in that region, with respect to the case of supercritical flow under relatively low free-atmosphere stratification.
- *The wind-farm wake region*: downwind of the wind-farm trailing edge, the absence of turbine thrust forces induces a streamwise acceleration of the flow and, due to conservation of mass, a downward flux of mean momentum. The farm wake flow, which is the result of the cumulative effect of all the turbine wakes in the wind farm, recovers momentum with increasing downwind distance until the wake is negligible and the flow resembles that upwind of the wind farm. Satellite measurements (Christiansen and Hasager 2005) and numerical simulations (Wu and Porté-Agel 2017) have shown that wind-farm wakes can have non-negligible effects on the surface-layer wind speed, which retains a 2% deficit at downwind distances from the farm in the range of 5–20 km, depending on the ambient stability and wind-farm size. Therefore, understanding and predicting these farm wake flows is essential to minimizing farm-to-farm interactions when large wind farms are to be deployed in proximity to each other. This is the case, for example, in the North Sea, where multiple wind farms are planned due to the combination of favourable high wind speeds and shallow water conditions (Christiansen and Hasager 2006).

### Fully-Developed (Infinite) Wind-Farm Flows

Even though the fully-developed wind-farm flow regime is seldom attained in existing wind farms due to their limited size, it has been extensively studied, particularly using analytical models (e.g., Frandsen 1992; Calaf et al. 2010; Abkar and Porté-Agel 2013) and large-eddy simulations (e.g., Calaf et al. 2010, 2011; Lu and Porté-Agel 2011; Abkar and Porté-Agel 2013; Yang et al. 2014a, b). This can be explained as follows: (a) the increasing likelihood of achieving fully-developed flow above the mega-size wind farms of the future; (b) the possibility of using more computationally-efficient numerical techniques (e.g., periodic boundary conditions in LES) with relatively small computational domains; and (c) the possibility of simplifying the flow governing equations and developing 1D top-down models for horizontally-averaged fully-developed wind-farm flows. These models can be used to predict power output in the fully-developed region of very large wind farms, and to parametrize their effects in large-scale weather and climate models.

**Fig. 12:**
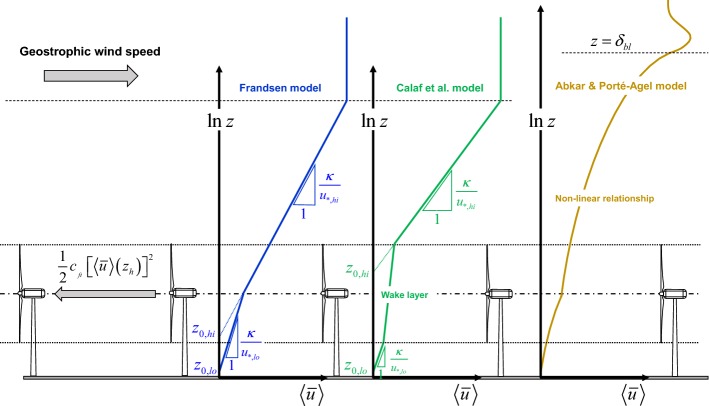
Different 1D models that predict the effective surface roughness for infinite wind farms. From left to right, the figure shows the models developed by Frandsen (1992), Calaf et al. (2010), and Abkar and Porté-Agel (2013), respectively

The most common approach used to describe fully-developed wind farms within 1D models is to represent them as an effective surface roughness. This method, commonly used for plant canopies, has been applied to wind-farm flows since the pioneering studies of Templin (1974) and Newman (1977), among others. However, Frandsen (1992), later elaborated in Frandsen et al. (2006), is the cornerstone of most of the fully-developed wind-farm flow models used nowadays. The Frandsen model is developed based on the following assumptions:

1. Vertical profiles of the horizontally-averaged wind speed in fully-developed wind farms can be split into two logarithmic layers; one below the turbine hub-height characterized by the friction velocity $u_{*,lo}$ and the roughness length $z_{0,lo}$, the other one above the hub height characterized by the friction velocity $u_{*,hi}$ and the roughness length $z_{0,hi}$. Thus,
  16a $$\begin{aligned} \langle {\bar{u}}\rangle _{lo}(z)&= \frac{u_{*,lo}}{\kappa }\ln \left( \frac{z}{z_{0,lo}}\right) \quad \text {for} \quad z<z_h, \end{aligned}$$
  16b $$\begin{aligned} \langle {\bar{u}}\rangle _{hi}(z)&= \frac{u_{*,hi}}{\kappa }\ln \left( \frac{z}{z_{0,hi}}\right) \quad \text {for} \quad z>z_h, \end{aligned}$$
  where $\langle \rangle$ denotes horizontal averaging and $\kappa$ is the von Kármán constant. The objective is to estimate $z_{0,hi}$, which is the effective roughness length for the logarithmic layer above the fully-developed wind farm. A schematic of this model, together with two other one-dimensional models developed in the literature, is shown in Fig. 12.
2. The vertical profile of the mean wind speed is continuous, i.e.,
  17 $$\begin{aligned} \langle {\bar{u}}\rangle _{hi}(z_h)=\langle {\bar{u}}\rangle _{lo}(z_h). \end{aligned}$$
3. Based on the balance of momentum and assuming that dispersive stresses are negligible, the difference between the values of the horizontally-averaged turbulent shear stress $\langle \overline{u'w'}\rangle$ corresponding to the two logarithmic layers is equal to the momentum loss caused by the turbines, i.e.,
  18 $$\begin{aligned} u_{*,hi}^2-u_{*,lo}^2=\left( -\langle \overline{u'w'}\rangle _{hi}\right) - \left( -\langle \overline{u'w'}\rangle _{lo}\right) =\frac{1}{2}c_{ft}\left[ \langle {\bar{u}}\rangle (z_h)\right] ^2, \end{aligned}$$
  where $c_{ft}=\uppi C_T/4s_xs_y$, and $s_x$ and $s_y$ are, respectively, the streamwise and spanwise turbine spacing normalized with the rotor diameter. It is worth mentioning that dispersive stresses are, in general, smaller than the horizontally-averaged shear stresses, but they are not negligible (Cal et al. 2010; Calaf et al. 2010). In particular, for aligned wind farms, they may comprise as much as 40$\%$ of the total shear stress due to the strong flow inhomogeneity in these wind farms (Markfort et al. 2012, 2017).

Solving Eqs. 16–18 to find $z_{0,hi}$ gives the Frandsen relation for the effective roughness length,

19 $$\begin{aligned} z_{0,\text {Frandsen}}=z_h\exp \left( -\frac{\kappa }{\sqrt{\frac{1}{2}c_{ft}+ \left[ \frac{\kappa }{\ln (z_h/z_{0,lo})}\right] ^2}}\right) . \end{aligned}$$

Based on the above work, Frandsen and Thøgersen (1999) also developed a simple relationship for the added turbulence intensity $\varDelta I$ in a fully-developed wind farm,

20 $$\begin{aligned} \varDelta I=\frac{a_1\sqrt{C_T}}{a_2\sqrt{C_T}+\sqrt{s_xs_y}}, \end{aligned}$$

where $a_1$ and $a_2$ are empirical coefficients, estimated to be 1.8 and 5, respectively.

Calaf et al. (2010) later employed LES to verify the presence of two logarithmic layers above and below the wind-turbine region. However, they found that, due to the increased mixing in the wind-turbine region, a third (middle) logarithmic layer with smaller velocity gradient exists at turbine height (i.e., from $z_h-d/2$ to $z_h+d/2$). The slope of this layer is determined based on a non-dimensional parameter, referred to as the wake eddy viscosity $\nu _{w}^*$. This method results in a modified version of the Frandsen model that is in better agreement with the LES data reported by Calaf et al. (2010). See also Meneveau (2012) for additional information on this model. The effective roughness length for a fully-developed wind farm given by this model is

21 $$\begin{aligned} \begin{aligned} z_{0,\text {C}}&=z_h\left( 1+\frac{d}{2z_h}\right) ^{\beta }\\&\quad \times \exp \left( -\left[ \frac{c_{ft}}{2\kappa ^2}+\left( \ln \left[ \frac{z_h}{z_{0,lo}}\left( 1-\frac{d}{2z_h}\right) ^{\beta }\right] \right) ^{-2} \right] ^{-1/2}\right) , \end{aligned} \end{aligned}$$

where $\beta =\nu _{w}^*/(1+\nu _{w}^*)$ and $\nu _{w}^*$ is roughly estimated as $28\sqrt{0.5c_{ft}}$. Note that the model of Calaf et al. (2010) (Eq. 21) is identical to that developed by Frandsen (Eq. 19) if $\nu _{w}^*\rightarrow 0$.

One-dimensional models of fully-developed wind-farm flows can also be used to estimate the spatially-averaged wind speed at hub height and, consequently, the power production as a function of wind-farm density (i.e., $s_x\times s_y$) and turbine loading (i.e., turbine thrust coefficient $C_T$) for infinite wind farms. Meyers and Meneveau (2012) used this method to calculate the power production of an infinite wind farm for different operating conditions. By considering the costs associated with wind turbines and land surface, they concluded that the optimal turbine spacing in infinite wind farms should be considerably higher than that commonly used in current wind farms. Stevens (2016), however, showed that the optimal turbine spacing for finite-size wind farms, obtained based on a method similar to that of Meyers and Meneveau (2012), is similar to that normally used in existing wind farms.

One of the limitations of the models developed by Frandsen (1992) and Calaf et al. (2010) originates from the fact that they are derived for the purely neutral ABL, which rarely occurs in reality. As discussed in Sect. 3.4.1, the neutrally-stratified ABL is often capped by a stably-stratified free atmosphere, which can affect the interaction of the ABL with large wind farms and their power production. With that in mind, Abkar and Porté-Agel (2013) modified the Frandsen model to account for the effect of free-atmosphere stability. This model is schematically shown in Fig. 12 and discussed more in Sect. 3.4.1.

Another limitation of these models is that they cannot differentiate between different layout configurations or different wind directions, as they depend only on the overall wind-farm density (i.e., number of turbines in a given area). Later studies, such as Yang et al. (2012) and Stevens et al. (2015), attempted to overcome this limitation by considering the wind-farm area only as the region influenced by turbine wakes (see Sect. 3.3.1 for more information on the latter study).

### Finite-Size Wind-Farm Flows

Owing to the fact that all wind farms are finite in size, flow distribution inside and above finite-size wind farms has been the subject of numerous wind-tunnel, field, and numerical studies in recent years (e.g., Corten et al. 2004; Frandsen et al. 2006; Barthelmie et al. 2007, 2009; Porté-Agel et al. 2011, 2013; Chamorro and Porté-Agel 2011; Markfort et al. 2012; Wu and Porté-Agel 2013, 2015; Newman et al. 2013; Creech et al. 2015; Hamilton et al. 2015; Munters et al. 2016; Na et al. 2016; Vanderwende et al. 2016; Andersen et al. 2017). The flow region ‘inside’ the wind farms (i.e., below the turbine top-tip height) is characterized by spatially-evolving low-speed flows with high turbulence intensity due to the cumulative effects of wind-turbine wakes. Both velocity deficit and enhanced turbulence intensity increase in the first few rows of turbine arrays, while their variation between the subsequent rows becomes progressively smaller. Several studies have shown that, for certain wind-farm layouts and wind directions, the flow inside some wind farms appears to asymptote to fully-developed conditions after the first several rows of wind turbines (e.g., Barthelmie et al. 2010; Chamorro et al. 2011; Markfort et al. 2012; Newman et al. 2013; Archer et al. 2013; Hamilton et al. 2015). However, as mentioned in Sect. 3.1, recent studies have shown that a much longer distance is required for the entire ABL flow to reach fully-developed conditions. This, in turn, leads to a relatively slow adjustment of the flow inside the wind-turbine region, compared to that observed in the entrance region of the wind farm. The flow adjustment distance, which depends on the incoming ABL flow properties (e.g., ABL height, atmospheric stability, wind speed and turbulence intensity), and wind-farm characteristics (power density and layout), can be two orders of magnitude larger than the incoming ABL height (Wu and Porté-Agel 2017). This implies that many large wind farms might never reach fully-developed conditions (e.g., Crespo et al. 1999a; Allaerts and Meyers 2017) and emphasizes the importance of studying the wind-farm flow-development region under different ABL conditions.

**Fig. 13:**
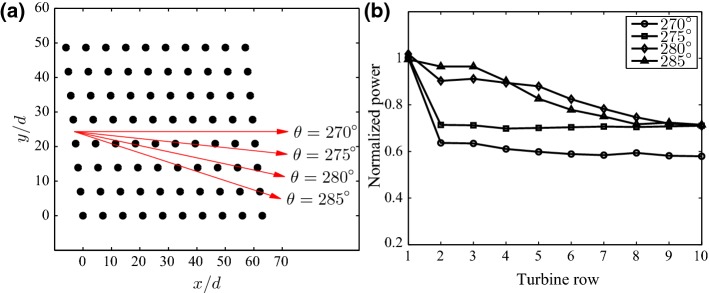
Horns Rev wind-farm power output in different wind direction scenarios: **a** schematic of the wind farm together with some selected wind direction angles $\theta$, **b** average power output of each row of turbines, normalized by the power of the first row. The data are obtained from the field measurements of Barthelmie et al. (2010)

Wind-farm layout has attracted a great deal of attention due to its strong impact on the flow development inside wind farms and, therefore, on their efficiency. Several recent wind-tunnel and LES studies have focused on the differences between two basic wind-farm layout configurations: (i) *aligned*, and (ii) *staggered* (e.g., Chamorro et al. 2011; Markfort et al. 2012; Wu and Porté-Agel 2013; Archer et al. 2013; Hamilton et al. 2015; Stevens et al. 2016a; Wu et al. 2019). Overall, it is found that inside staggered wind farms, wind turbines are subject to relatively smaller wake effects (power losses and fatigue loads). This can be explained by the relatively larger effective distance (in the wind direction) between turbines in this configuration, which allows turbine wakes to attain lower velocity deficits and turbulence intensities when they interact with downwind turbines. It is also found that wake-induced power losses display a more gradual change with downstream distance in staggered wind farms compared with aligned ones. Moreover, even though vertical kinetic-energy entrainment is less localized in staggered wind farms (Stevens et al. 2016a), they benefit, in general, from more effective total vertical entrainment (Hamilton et al. 2015).

**Fig. 14:**
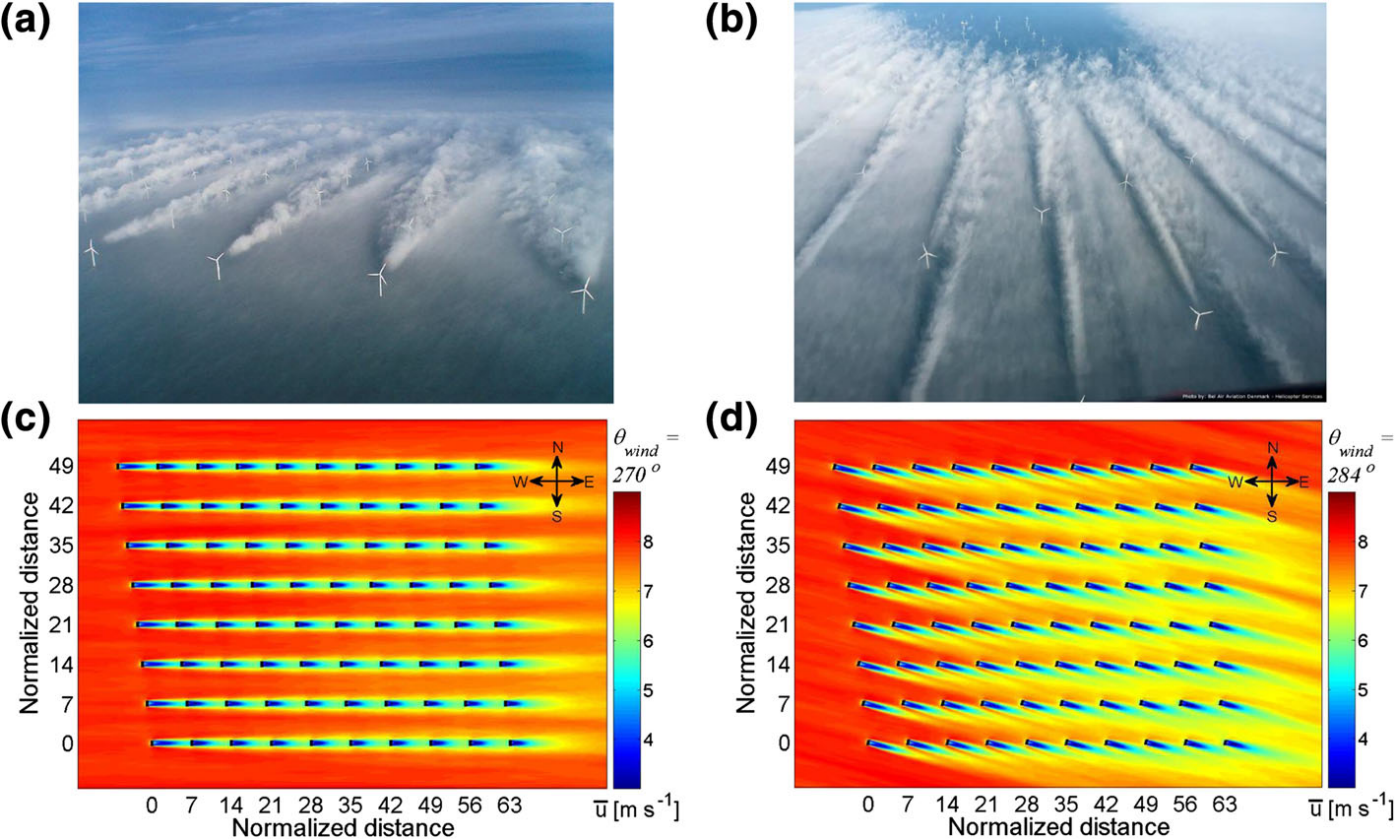
**a** Full-wake conditions seen in the photo taken from Horns Rev wind farm (Courtesy: Vattenfall. Photographer is Christian Steiness), **b** Partial-wake conditions seen in the photo taken from Horns Rev 2 wind farm (figure taken from Hasager et al. 2017, in accordance with the Creative Commons Attribution (CC BY) license). Contour plot of the simulated time-averaged streamwise velocity component at Horns Rev wind farm on a horizontal plane at hub level for incoming wind directions of **c**$270^{\circ }$ (full-wake conditions) and **d**$284^{\circ }$ (partial-wake conditions). Distances are normalized by the turbine rotor diameter $d = 80$ m (**c**, **d** taken from Porté-Agel et al. 2013, in accordance with the Creative Commons Attribution (CC BY) license)

**Fig. 15:**
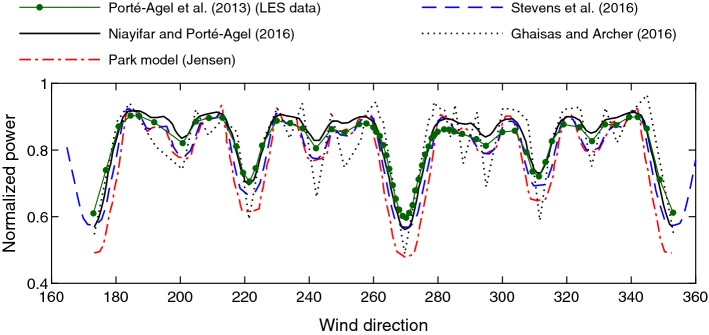
Distribution of the normalized Horns Rev wind-farm power output for different wind directions. The predictions of different wind-farm analytical models are compared with LES data

It should be mentioned that staggered and aligned configurations are only two possible, not necessarily the most common, layouts that can be found in wind farms. Indeed, for a given wind-farm configuration, the *effective layout* of the wind farm (with respect to the incoming flow) changes with changing wind direction. Several field studies (e.g., Barthelmie et al. 2005, 2007, 2009, 2010; Gaumond et al. 2013) have shown that wind direction and its variability have profound effects on wind-farm power output. Figure 13 shows the layout of the Horns Rev wind farm, together with the simulated normalized power output as a function of wind-turbine row for four selected wind directions (Barthelmie et al. 2010). Porté-Agel et al. (2013) performed LES to study how changing wind direction affects the performance of the same wind farm. The time-averaged streamwise velocity component at hub level for two of those wind directions is shown in Fig. 14. Finally, Fig. 15 shows the variation of the normalized total power output from the Horns Rev wind farm as a function of wind direction. As shown, wind-farm power reaches its minimum value for a wind direction of $270^{\circ }$, corresponding to the aligned case with the smallest effective distance between turbines, and it shifts to its maximum value with a relatively small change in wind direction ($270^{\circ }\pm 10^{\circ }$), for which the effective distance between turbines is maximum. Stevens et al. (2014) reported a similar value for the optimum angle (around $10^{\circ }$) between the wind direction and the turbine columns of a wind-farm array. This strong sensitivity of wind-farm power output to small variations of the wind direction should be taken into account for the optimal control and grid integration of wind farms.

#### Analytical Modelling of Finite-Size Wind-Farm Flows

The most common approach to analytically model finite-size wind-farm flows is to model each turbine wake using one of the analytical models presented in Sect. 2.2.3, while applying superposition methods to account for the interaction among multiple wakes. Since the pioneering study of Lissaman (1979), different superposition methods have been proposed in the literature (Crespo et al. 1999b). A summary of the different methods available to estimate velocity at a given position $\mathbf{X }=(x,y,z)$ in a wind farm is given in Table 1. The velocity at each position is a function of the velocity deficit induced by all the upwind turbines (from $i=1$ to *n*) whose wakes affect the flow in that location. The differences among the methods presented in Table 1 originate from the use of:

- *Different superposition principles*: linear superposition of velocity deficit (Lissaman 1979; Niayifar and Porté-Agel 2016), or linear superposition of energy deficit (Katić et al. 1986; Voutsinas et al. 1990a).
- *Different definitions of the velocity deficit caused by thei*th *turbine*: it can be defined either with respect to the incoming boundary-layer flow speed $u_{\infty }$ (Lissaman 1979; Katić et al. 1986), or with respect to the incoming flow speed for that turbine $u_{in,i}$ (Voutsinas et al. 1990a; Niayifar and Porté-Agel 2016).

**Table 1:** Different superposition techniques used in the literature to model wake interactions in wind farms

Superposition method	Definition
Lissaman (1979)	$u(\mathbf{X })=u_{\infty }-\sum \nolimits _{i=1}^{n}\varDelta u_i(\mathbf{X }),$ where $\varDelta u_i(\mathbf{X })=u_{\infty }-u_i(\mathbf{X })$
Katić et al. (1986)	$u(\mathbf{X })=u_{\infty }-\sqrt{\sum \nolimits _{i=1}^{n}\varDelta u_i^2(\mathbf{X })},$ where $\varDelta u_i(\mathbf{X })=u_{\infty }-u_i(\mathbf{X })$
Voutsinas et al. (1990a)	$u(\mathbf{X })=u_{\infty }-\sqrt{\sum \nolimits _{i=1}^{n}\varDelta u_i^2(\mathbf{X })},$ where $\varDelta u_i(\mathbf{X })=u_{in,i}-u_i(\mathbf{X })$
Niayifar and Porté-Agel (2016)	$u(\mathbf{X })=u_{\infty }-\sum \nolimits _{i=1}^{n}\varDelta u_i(\mathbf{X }),$ where $\varDelta u_i(\mathbf{X })=u_{in,i}-u_i(\mathbf{X })$

The most common analytical wake model for wind farms is the so-called Park model, used extensively in the literature (Crespo et al. 1999b; Barthelmie et al. 2009; Barthelmie and Jensen 2010) and in industry-standard software such as WAsP (Wind Atlas Analysis and Application Program) (Barthelmie et al. 2005). This model is based on the Jensen analytical wake model (Jensen 1983), along with the wake superposition approach suggested by Katić et al. (1986). Predictions of the power output from the Horns Rev wind farm by this model are shown in Fig. 15 for different wind-direction angles, and compared with LES data reported by Porté-Agel et al. (2013). For the Park model predictions shown in Fig. 15, a constant linear wake growth rate equal to 0.04 is used according to the semi-empirical formula suggested by Frandsen (1992) (Eq. 6).

As mentioned in Sect. 2.2.3, in order to use analytical wake models, a priori estimation of the wake growth rate is needed. The proper estimation of the wake growth rate is especially important and challenging in the prediction of wind-farm flows, as it is influenced by the spatial variation of turbulence intensity inside the wind farm. In this respect, wakes of turbines deep inside wind farms are expected to grow faster than the wakes of those in the first row. To account for variable wake growth rates in wind farms, Stevens et al. (2015, 2016b) coupled the Park model with the 1D model developed by Calaf et al. (2010), discussed in Sect. 3.2. Based on this coupled model, the wake growth rate exponentially increases from the value suggested by the Frandsen (1992) relation (Eq. 6) at the wind-farm entrance region to an asymptotic value deep inside the wind farm (i.e., fully-developed region). The asymptotic value of the wake growth rate is obtained through an iterative procedure. The predicted values of this model for the normalized power generated by the Horns Rev wind farm are also shown in Fig. 15.

As discussed in Sect. 2.2.3, the use of the top-hat distribution to describe velocity-deficit profiles is an overly simplified assumption that may result in unrealistic predictions, especially in the case that multiple wakes interact in large wind farms. To overcome this limitation, recent studies (e.g., Niayifar and Porté-Agel 2016; Parada et al. 2017) have employed the model developed by Bastankhah and Porté-Agel (2014b), which assumes a self-similar Gaussian distribution for the wake velocity-deficit profiles, to predict wind-farm flows. Niayifar and Porté-Agel (2016) employed a new approach to superpose wakes of different turbines in wind farms (see Table 1). A variable wake growth rate *k* changing with the incoming turbulence intensity for each turbine was also used based on Eq. 15. This model predictions of the power generated by the Horns Rev wind farm are also shown in Fig. 15.

As mentioned earlier, simple analytical models are particularly useful for the purpose of wind-farm layout optimization. In this context, analytical models have been extensively implemented in combination with different optimization techniques with the ultimate goal of optimizing wind-farm performance. The interested reader is referred to Grady et al. (2005), Marmidis et al. (2008), Kusiak and Song (2010), González et al. (2010), González et al. (2011), Chen et al. (2013), Wagner et al. (2013), Chowdhury et al. (2013), and Gebraad et al. (2014), amongst others.

### Thermal Effects

The aforementioned studies of wind-farm flows assume purely neutral ABL conditions, which rarely occur in reality. Indeed, even in situations when the ABL itself is close to neutral (i.e., constant mean potential virtual temperature throughout most of the boundary layer), the free atmosphere is often thermally stratified. Moreover, surface-layer stability can strongly modulate the interaction between wind farms and the ABL. The following is a summary of recent research on thermal effects in wind-farm flows.

#### Free-Atmosphere Stratification

The conventionally-neutral ABL is characterized by neutrally-stratified flow (constant potential temperature), capped by a thermally-stratified free atmosphere (Zilitinkevich and Esau 2002). The potential temperature gradient (lapse rate) in the free atmosphere $\varGamma$ is typically constant with height and falls in the range of 1–10 K $\text {km}^{-1}$ (Sorbjan 1996). Between the boundary layer and the free atmosphere lies a relatively thin and more strongly stratified layer commonly referred to as the inversion layer, even if in some cases it might not be a thermal inversion.

The interaction of the conventionally-neutral ABL and very large wind farms has been investigated by, e.g., Abkar and Porté-Agel (2013, 2014) and Allaerts and Meyers (2015, 2017). Churchfield et al. (2012a) and Archer et al. (2013) also performed simulations of wind farms in the conventionally-neutral ABL.

Abkar and Porté-Agel (2013, 2014) studied the effect of the value of $\varGamma$ on the interaction between a fully-developed (infinite) wind farm and the conventionally-neutral ABL, concluding that increasing free-atmosphere stratification ($\varGamma$) leads to a reduction of the entrainment of kinetic energy from the free atmosphere. This, in turn, causes the ABL depth to become shallower and the wind-farm power output to decrease. Abkar and Porté-Agel (2013) proposed a 1D model to predict the effect of free-atmosphere stratification on the mean wind velocity profile, the ABL depth, and the power output of the wind farm. The model is based on the one developed by Frandsen (1992) and discussed in Sect. 3.2. The main difference is that, unlike Frandsen’s model, which is derived for a purely neutral ABL, the model of Abkar and Porté-Agel (2013) includes the effect of the stably-stratified free atmosphere, which leads to vertical profiles of horizontally-averaged wind speed below and above hub height that are different from those given by Eq. 16. In this case, $\langle {\bar{u}}\rangle _{lo}(z)$ and $\langle {\bar{u}}\rangle _{hi}(z)$ are given by

22a $$\begin{aligned} \langle {\bar{u}}\rangle _{lo}(z)&= \frac{u_{*,lo}}{\kappa }\ln \left( \frac{z}{z_{0,lo}}\right) +a_uNz \quad \text {for} \quad z<z_h, \end{aligned}$$

22b $$\begin{aligned} \langle {\bar{u}}\rangle _{hi}(z)&= \frac{u_{*,hi}}{\kappa }\ln \left( \frac{z}{z_{0,hi}}\right) +a_uNz \quad \text {for} \quad z>z_h, \end{aligned}$$

where $a_u$ is an empirical constant with the value around 0.3, and *N* denotes the Brunt–Väisälä frequency that mainly depends on the lapse rate $\varGamma$. The flow continuity at hub height (Eq. 17) and the balance of momentum (Eq. 18) for the velocity profiles expressed by Eq. 22 yield

23 $$\begin{aligned} u_{*,hi}= & {} \left[ \left( \frac{\kappa }{\ln \left( z_h/z_{0,lo}\right) } \left( \langle {\bar{u}}\rangle (z_h)-a_uNz_h\right) \right) ^2+\frac{1}{2}c_{ft} \left[ \langle {\bar{u}}\rangle (z_h)\right] ^2\right] ^{1/2}, \end{aligned}$$

24 $$\begin{aligned} \langle {\bar{u}}\rangle (z_h)= & {} G-\frac{u_{*,hi}}{\kappa }\ln \left( \frac{\delta _{bl}}{z_h}\right) -a_uN\left( \delta _{bl}-z_h\right) , \end{aligned}$$

where the boundary-layer thickness $\delta _{bl}$ is given by (Zilitinkevich and Esau 2002)

25 $$\begin{aligned} \delta _{bl}=C_R\left( 1+C_N\frac{N}{|f|}\right) ^{-1/2}\frac{u_{*,hi}}{f}+z_h+ \frac{d}{2}, \end{aligned}$$

where $C_R$ and $C_N$ are empirical constants and *f* is the Coriolis parameter. The system of Eqs. 23–25 can be numerically solved to yield the values of $u_{*,hi}$, $\langle {\bar{u}}\rangle (z_h)$ and $\delta _{bl}$. The value of the wind-farm effective roughness length can be then found by solving Eq. 22 for $z_{0,hi}$ at $z=z_h$. A feature of this model is that it can also predict the increase of the ABL thickness caused by the presence of a very large wind farm.

Allaerts and Meyers (2015) studied the role of changing both the free-atmosphere stratification and the inversion-layer characteristics on the interaction between the conventionally-neutral ABL and an infinite wind farm using LES. To do that, they imposed the height, thickness, and strength of the inversion layer, instead of letting it develop as previously done in the aforementioned study. In particular, they investigated the effect of the inversion-layer strength $\varDelta \theta _{I}$ and the base height of the inversion layer (i.e., the height of the ABL), and found that, with increasing inversion-layer strength and decreasing ABL height, the power output of the farm decreases. They also proposed a simple analytical model to obtain the wind-farm power output for the fully-developed regime as a function of the ABL height and the Rossby number.

Investigation of the interaction of wind farms with the conventionally-neutral ABL has not been limited to the infinite wind-farm case, and the more realistic finite-size wind-farm case has also been studied (Allaerts and Meyers 2017; Wu and Porté-Agel 2017). Unlike the infinite wind-farm case, the flow through a finite-size wind farm evolves in the streamwise direction, such that an IBL develops above the wind turbines. The IBL development, in turn, causes vertical displacement of the flow, which can lead to gravity waves. A significant consequence of these gravity waves is that they induce pressure gradients across the wind farm (Allaerts and Meyers 2017; Wu and Porté-Agel 2017; Allaerts and Meyers 2018). If $\varGamma$ is sufficiently large, such that the Froude number of the flow is less than one (subcritical flow), then gravity waves can propagate upwind, leading to the creation of an adverse pressure gradient zone in the induction region, and a favourable pressure gradient zone in the exit region of the wind farm. The gravity-wave-induced deceleration of the flow in the induction region leads to a reduction in the energetic performance of the first turbine rows with respect to the cases in which gravity waves are not present. In contrast, the favourable pressure gradient in the exit region leads to a flow acceleration and, consequently, an increase in the power output of the turbines in that region (Wu and Porté-Agel 2017).

#### Surface-Layer Stability Effects

Consistent with the single turbine case (Sect. 2.3), surface-layer thermal stability can affect wind-farm performance in several ways. For example, the mean wind shear and flow speed at turbine level, which are often larger under stable conditions, can lead to differences in the available energy and also in the power output from the wind farm. Furthermore, surface-layer stability has a strong influence on the power losses induced by turbine-wake flows. It has been shown that the efficiency of a wind farm (which is inversely related to the power losses due to wake effects) is higher in convective regimes and lower in stable ones (Barthelmie and Jensen 2010; Hansen et al. 2012; Schepers et al. 2012; Abkar et al. 2016). The reason for this trend is the fact that, as discussed in Sect. 2.3 for single turbine wakes, the turbulence intensity of the ABL flow and, consequently, the wake recovery rate are inversely related to thermal stability (Christiansen and Hasager 2005; Barthelmie and Jensen 2010; Hansen et al. 2012; Schepers et al. 2012; Abkar et al. 2016).

Emeis (2010) and Peña and Rathmann (2014) extended the Emeis and Frandsen (1993) and Frandsen (1992) models, respectively, to take into account surface-layer stability effects in the estimation of the wind-speed reduction and energetic efficiency of infinite-size wind farms.

It should be mentioned that the interaction of relatively large wind farms with the thermally-stratified ABL leads to modifications of the surface momentum and heat fluxes inside the wind farm. It can also affect the entrainment fluxes from the free atmosphere if the wind farm is large enough. Therefore, special care has to be paid to considering both effects when studying and modelling the two-way interaction between wind farms and thermally-stratified ABLs. This is further discussed in Sect. 3.5.

#### Diurnal Cycle

Although studying wind farms under stationary (or quasi-steady) thermal stability conditions (i.e., purely stable, unstable, neutral or conditionally neutral) allows isolation of the effect of different thermal stabilies, the non-stationarity of the ABL flow plays also an important role on the interaction between wind farms and the ABL. Particularly important is the effect of the ubiquitous non-stationarity associated with the diurnal cycle, which has been investigated in several recent (mainly LES) studies (Schepers et al. 2012; Fitch et al. 2013; Abkar et al. 2016; Rodrigo et al. 2017; Sharma et al. 2017).

An important feature of wind farms in diurnal cycles is the history effects. In other words, the wind-farm performance at a specific point in time in a diurnal cycle is influenced by the history of the ABL before that point in time. For example, in the morning and evening transitions, even if the background ABL can be considered as near-neutral in both cases (based on, for instance, the vertical profile of the mean potential temperature or the value of the Obukhov length), the wind-farm power deficit due to wake effects can be rather different. This is because wake recovery and, thus, power losses are influenced by the overall flow characteristics (e.g., flow depth, range of turbulence scales, or wind direction change with height), which depend on the previous stable (morning transition) or unstable (evening transition) regimes (Abkar et al. 2016).

On the comparison between the daytime and night-time performance of wind farms, one can mention two important differences: (1) the difference in recovery rate of the wakes due to stability, (2) the difference in the vertical profile of the incoming wind speed experienced by the wind farm. Considering the first difference, aligned with the discussions of the previous subsection, it has been widely shown that the power deficit due to turbine wakes is considerably smaller during the daytime (CBL) than during night-time (SBL) (Schepers et al. 2012; Fitch et al. 2013; Abkar et al. 2016; Sharma et al. 2017). This is due to the fact that turbine wakes recover faster during the day, owing to the relatively higher atmospheric turbulence intensity, compared with the night-time. The large changes in wind direction with height often observed during the late night, resulting from the effect of the Coriolis force on the relatively shallow SBL, can also have an effect on the wake shape and, consequently, on the wake-induced power losses.

Regarding the differences in the vertical profile of the incoming wind speed experienced by the wind farms during the daytime and night-time, a key factor is the presence and location of the low-level jet (LLJ), which can develop at night. The LLJ forms in and above the SBL when a maximum in the mean velocity profile emerges at the top of the boundary layer (Blackadar 1957). This maximum speed is super-geostrophic, and its height from the ground (i.e., the height of the LLJ) is usually 100–300 m (Stull 2012), although values as low as 50 m have been reported (e.g. Song et al. 2005). Below the LLJ, the SBL is characterized by high shear, while laminar free-atmosphere flow is commonly found above. The height of the LLJ depends on the strength of the stratification of the SBL, in a way that increasing stability leads to lowering of the LLJ (Banta 2008; Zhou and Chow 2012; Huang and Bou-Zeid 2013). Considering the above-mentioned range of LLJ heights, the LLJ can be either above the turbine rotor, within the rotor-disk height range, or even in extreme cases, below the rotor. When the LLJ forms in the rotor-disk region, it provides large available power for extraction by the turbines (Fitch et al. 2013; Abkar et al. 2016; Sharma et al. 2017), while the recovery rate of the wake is reduced because of the non-turbulent flow above the LLJ (Bhaganagar and Debnath 2015). It is worthwhile to mention that, in the interaction with large wind farms, the LLJ does not remain unchanged. In fact, as a result of that interaction, the LLJ can be either eliminated (Lu and Porté-Agel 2011; Fitch et al. 2013) or shifted upwards (Abkar et al. 2016; Sharma et al. 2017).

**Fig. 16:**
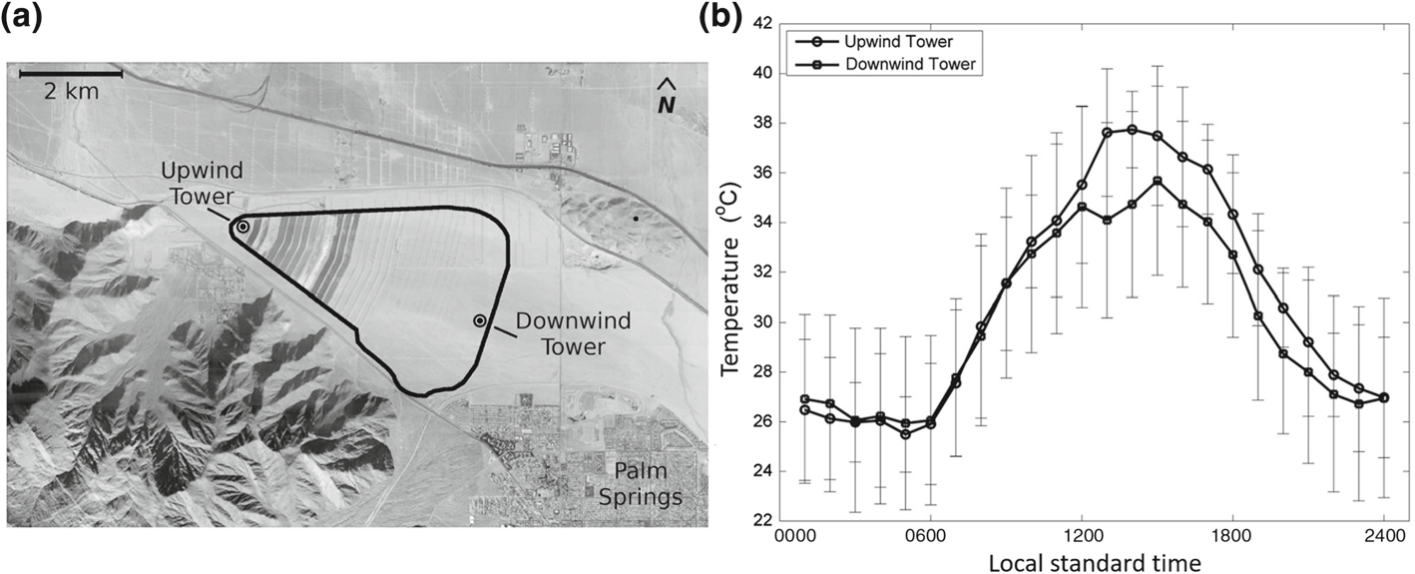
Effect of wind farms on surface air temperature. **a** Google Earth image of the wind farm at San Gorgonio, California, USA, showing the wind-farm boundary in 1989. **b** Observed near-surface air temperature at the wind-farm Figure reprinted from Baidya Roy and Traiteur (2010) with the automatic permission of National Academy of Sciences for the reuse of original PNAS figures in review articles

Depending on all the aforementioned factors, the differences between the wind-farm power output during daytime and night-time can change significantly; for example, Fitch et al. (2013) reported a higher power output during the night-time, while Abkar et al. (2016) and Sharma et al. (2017) reported a higher power output during the daytime. Furthermore, the power density (power per unit surface area) of the wind farm can be a factor in this regard. For instance, Sharma et al. (2017) have shown that reducing the power density of a wind farm can lead to an increase in the power output at night, as a result of the smaller upward deflection of the LLJ.

### Effect of Wind Farms on Local Meteorology

In a pioneering study, Baidya Roy et al. (2004) demonstrated that wind farms can have a significant impact on near-surface air temperature (see Fig. 16). Later experimental and numerical studies have confirmed that wind farms, through their interaction with the ABL, can potentially affect local meteorology (e.g., Baidya Roy and Traiteur 2010; Lu and Porté-Agel 2011; Baidya Roy 2011; Zhou et al. 2012; Fitch et al. 2013; Smith et al. 2013; Cervarich et al. 2013; Zhou et al. 2013; Rajewski et al. 2013; Calaf et al. 2014; Armstrong et al. 2014; Lu and Porté-Agel 2015; Xia et al. 2016; Sharma et al. 2017; Moravec et al. 2018; Adkins and Sescu 2018; Siedersleben et al. 2018). Particularly interesting in this respect is the ability of large wind farms to modify surface fluxes of momentum and scalars (e.g., temperature and moisture) as well as near-surface wind speed and scalar concentration. Regardless of atmospheric stability, the near-surface wind speed and the kinematic surface momentum flux ($-u_*^2$) decrease as a result of the extraction of momentum by the wind turbines (e.g., Lu and Porté-Agel 2011; Fitch et al. 2013; Lu and Porté-Agel 2015; Sharma et al. 2017). The changes in near-surface scalar concentration and surface scalar flux are less straightforward and different studies have reported different trends. Below we provide a summary of the main results reported to date.

The changes in near-surface temperature induced by wind farms are found to be more pronounced in the nocturnal SBL (Zhou et al. 2012; Fitch et al. 2013; Smith et al. 2013; Zhou et al. 2013; Lu and Porté-Agel 2015; Xia et al. 2016). At night-time (stable regime), it is well established that the presence of a wind farm leads to a temperature increase (warming) near the surface (Baidya Roy et al. 2004; Baidya Roy and Traiteur 2010; Lu and Porté-Agel 2011; Baidya Roy 2011; Zhou et al. 2012; Fitch et al. 2013; Smith et al. 2013; Cervarich et al. 2013; Zhou et al. 2013; Xia et al. 2016; Sharma et al. 2017). Conversely, during the daytime (convective regime), the change in near-surface temperature has been shown to be relatively weaker (Zhou et al. 2012; Smith et al. 2013; Cervarich et al. 2013; Zhou et al. 2013; Xia et al. 2016). Lu and Porté-Agel (2015) showed with LES that near-surface and land-surface temperatures rise slightly as a result of the presence of very large wind farms. It should be noted that Baidya Roy et al. (2004), Baidya Roy and Traiteur (2010), and Baidya Roy (2011) reported a cooling effect during convective conditions, based on simulations with a relatively low resolution mesoscale weather model. This seemingly contradictory result was addressed experimentally later by Zhou et al. (2012) who, using satellite data, showed that large wind farms increase slightly the land-surface temperature during the daytime.

In order to understand the effect of wind farms on the surface heat flux, it is useful to recall that the kinematic surface heat flux can be expressed as $q_s=-u_* \theta _*$, where $\theta _*$ is a temperature scale related to the gradient of the potential temperature above the surface. Hence, even though wind farms always tend to reduce the friction velocity $u_*$, which would reduce surface scalar fluxes, the relative change in $|q_s|$ is also affected by the variation in the magnitude of $\theta _*$. LES studies have shown a considerable reduction in the magnitude of the surface heat flux induced by fully-developed wind farms in the SBL (Lu and Porté-Agel 2011), while a much smaller reduction in $|q_s|$ is observed in the CBL (Lu and Porté-Agel 2015). Sharma et al. (2017) also showed a decrease in $|q_s|$ during the morning hours. Zhang et al. (2013a), in their wind-tunnel study, reported also a slight net reduction in $|q_s|$ for the CBL and showed that the spatial distribution of $|q_s|$ is heterogeneous over the surface, and is dependent on the wind-farm layout (i.e., staggered and aligned). A similar heterogeneous surface-flux distribution was found by Lu and Porté-Agel (2015) using LES. In contrast with some of the above studies, Baidya Roy et al. (2004) and Fitch et al. (2013), using mesoscale simulations, reported an increase in $|q_s|$ in the stable regime. Sescu and Meneveau (2015) found that $|q_s|$ increases in stable and slightly unstable regimes, while it slightly decreases in strongly unstable conditions.

The aforementioned differences in the effect of wind farms on surface heat flux and near-surface temperature reported by different numerical studies are likely due to differences in the numerical method (e.g., RANS vs. LES), grid resolution, wind-turbine parametrization, turbulence model and surface boundary condition. In LES, the turbines are normally modelled with standard actuator disk models (ADM-NR) (Jiménez et al. 2010), rotating actuator disk models (ADM-R) (Wu and Porté-Agel 2011), or actuator line models (ALM) (Shen and Sørensen 2002). Of those, only the last two are able to capture wake rotation effects. In numerical weather prediction models, wind turbines are parametrized as sinks of momentum and sources of turbulence, averaged over large areas (typically several $\hbox {km}^2$) corresponding to their relatively coarse spatial resolution. Additionally, the surface thermal boundary condition can also be a potential source of errors. In LES, this consists of specifying the surface temperature, fixing the surface heat flux, or dynamically computing both by coupling the flow simulation with a 3D soil heat equation model through the surface energy balance. In future LES studies of wind-farm flows, this last approach could be extended to include vegetation effects on heat and moisture fluxes using a soil–vegetation–atmosphere transfer model.

Sescu and Meneveau (2015) developed a single-column model to estimate the effect of infinite wind farms on the surface heat flux and vertical profiles of velocity and temperature in different atmospheric stability conditions. Their approach is a generalization of the previous models of Frandsen (1992), Calaf et al. (2011), Emeis (2010), and Abkar and Porté-Agel (2013).

Besides the effect of wind farms on local meteorology, recent studies using general circulation models (GCMs) have shown that extensive installation of wind farms over vast areas could produce non-negligible effects on the atmospheric flow at synoptic and continental scales (Keith et al. 2004; Kirk-Davidoff and Keith 2008; Barrie et al. 2010; Wang and Prinn 2010; Jacobson and Archer 2012; Adams and Keith 2013).

#### Wind-Farm Parametrization in Weather and Climate Models

Depending on the scale and resolution of the large-scale atmospheric model, two main types of wind-farm parametrizations are used. In the first type, the wind farm is considered as an increased surface roughness length. This type of parametrization is normally employed in GCMs, where the vertical resolution of the grid is so coarse that the lowest grid level falls above the height of the turbines (Ivanova and Nadyozhina 2000; Keith et al. 2004; Kirk-Davidoff and Keith 2008; Barrie et al. 2010; Wang and Prinn 2010, 2011). Specifically, the models described in Sect. 3.2 are commonly used to determine the effective surface roughness that takes into account the presence of the wind farm in GCMs. In the second type of wind-farm parametrizations, the wind farm is considered as an elevated sink of momentum and, at the same time, as a source of TKE. They are usually employed in mesoscale numerical weather prediction (NWP) models, where the vertical resolution of the grid is such that the lowest grid level falls below the turbine hub height. A summary of parametrizations of the second type is provided below.

In NWP models, the horizontal resolution ($\varDelta x$ and $\varDelta y$ ) is such that several turbines can be located within the horizontal extent of one grid cell. However, the vertical resolution ($\varDelta z$), as mentioned above, is fine enough to cover the rotor area with one or more grid levels. We first consider a control volume that entirely encompasses the turbine rotor area. To model the turbines as momentum sinks, one can adopt either a direct or an indirect approach. In the direct approach (Abkar and Porté-Agel 2015b), the induced force of each turbine is directly written as $F_t=\frac{1}{2} C_T \rho U^2A_r$, where *U* is a reference flow speed and $A_r$ is the rotor area. As the momentum equations are normally written with the dimension of force per unit mass, the momentum sink term for a control volume, which encompasses the turbine rotor, takes the form , where  is the volume of the control volume. In the indirect approach (Baidya Roy et al. 2004; Blahak et al. 2010; Fitch et al. 2012), the turbine is regarded as a sink of kinetic energy. The turbine extracts kinetic energy of the flow at a rate $\dot{E}_t=\frac{1}{2} C_{{ KE }} \rho U^3 A_r$, where $C_{{ KE }}$ is the fraction of the available kinetic energy that is extracted by the turbine. On the other hand, the kinetic energy of the control volume, assuming that *U* is the average wind speed in the volume, can be written as . The rate of change of  due to the presence of the turbine is . Equating  and $E_t$ results in . This term is the momentum tendency, which is treated the same as $f_t$ and can be regarded as the momentum sink in the momentum equation.

In Baidya Roy et al. (2004), $C_{{ KE }}$ is taken to be equal to the turbine’s power coefficient $C_P$. For the TKE source term, they use a constant value ($\beta$). This constant value is, in turn, considered as an additional kinetic energy sink, to conserve energy. In Blahak et al. (2010), $C_{{ KE }}$ is set equal to $C_a=C_P/\eta _{em}$, where $\eta _{em}$ is a loss factor due to mechanical and electrical losses. They consider the TKE source term to be a constant fraction ($\alpha$) of ${\dot{{ E }}}_t$ and, again to conserve energy, subtract the same amount from the kinetic energy. Finally, Fitch et al. (2012) assume that the turbine extracts a fraction $C_T$ of the available kinetic energy from the flow (i.e., $C_{{ KE }}=C_T$) and from this extracted energy a fraction $C_P$ is converted to electricity and a fraction $(C_T-C_P)$ is converted to TKE. In all three models, the flow speed in the computational grid cell is used to evaluate the aforementioned sink and source.

**Fig. 17:**
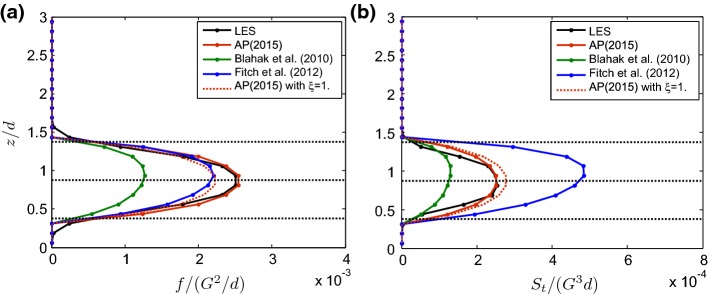
Vertical profiles of **a** the drag forces induced by the turbines (i.e., *f*), and **b** added TKE induced by the turbines (i.e., $S_t$) for a typical staggered infinite wind farm [see case $s7 \times 7$ in Abkar and Porté-Agel 2015b, referred to in the figure as AP(2015)]. Both *f* and $S_t$ are calculated according to Table 2 and are normalized by an appropriate combination of geostrophic wind speed *G* and rotor diameter *d* Figure reprinted from Abkar and Porté-Agel (2015b) with the permission of AIP Publishing

Abkar and Porté-Agel (2015b), on the other hand, adopt a different approach. First of all, they use the direct approach to calculate the momentum sink. Second, they analytically derive the TKE source term based on the resolved-scale TKE budget equation. Moreover, instead of the grid-cell flow speed, they use a modified value (computed with a correction factor, denoted $\xi$ in Table 2) to account for the difference between the grid-cell flow speed, the undisturbed flow speed ($U_{\infty }$) and the flow speed at the turbine rotor. Through this modification, the effect of turbine layout can also be taken into account. A comparison of the results of some of the aforementioned models to those of high-resolution LES is presented in Fig. 17.

The above-mentioned models are summarized in Table 2. In this table, $(u_i)_k$ is the *i*th component ($i=1,2$) of the flow velocity in a grid cell at the *k*th vertical level, the subscript *r* indicates the resultant horizontal flow speed, the subscript *h* means the quantity at hub-height level, $n_t=N_t/(\varDelta x \varDelta y)$, where $N_t$ is the number of wind turbines in a specific grid cell, $A_k$ is the area of the rotor segment that is trapped in the grid cell, $(z_{k+1} - z_k)$ is the thickness of the grid cell, *a* is the induction factor of the turbine, which can be calculated as $a=0.5(1-\sqrt{1-C_T})$, and $\xi =U_{\infty }/(u_r)_h$.

**Table 2:** Summary of wind farm parametrizations

Model	Momentum sink	TKE source
Baidya Roy et al. (2004)	$f_i=n_t \dfrac{[\dfrac{1}{2} C_P (u_r)_h (u_i)_h +\beta ] A_k }{z_{k+1}-z_k}$	$S_{t}=n_t\dfrac{\beta {(u_r)_h} A_k }{z_{k+1}-z_k}$
Blahak et al. (2010)	$f_i=n_t (1+\alpha )\dfrac{\dfrac{1}{2} C_a (u_r)_k (u_i)_k A_k }{z_{k+1}-z_k}$	$S_{t}=n_t\alpha \dfrac{\dfrac{1}{2} C_a {(u_r)_k}^3 A_k }{z_{k+1}-z_k}$
Fitch et al. (2012)	$f_i=n_t\dfrac{\dfrac{1}{2} C_T (u_r)_k (u_i)_k A_k }{z_{k+1}-z_k}$	$S_{t}=n_t\dfrac{\dfrac{1}{2} (C_T-C_P) {(u_r)_k}^3 A_k }{z_{k+1}-z_k}$
Abkar and Porté-Agel (2015b)	$f_i=n_t\dfrac{\dfrac{1}{2} C_T \xi ^2 (u_r)_h(u_i)_h A_k }{z_{k+1}-z_k}$	$S_{t}=n_t\dfrac{\dfrac{1}{2} C_T \xi ^2 {(u_r)_h}^3 A_k (1-(1-a)\xi ) }{z_{k+1}-z_k}$

## Topography

Unlike offshore wind farms, an onshore wind farm has a high chance of being located in non-flat terrain, i.e., topography. In this context, it is readily noticeable that most of the research on aerodynamics of wind farms is limited to turbines on flat surfaces (e.g., see the previous sections). Although there is deep and extensive literature on ABL flows over topography, the combination of wind turbines and topography still has much room for investigation.

Measurements of wind-turbine wakes on topography began in the early 1990s with the wind-tunnel study of Taylor and Smith (1991). Later, other measurements were performed both in the wind tunnel and in the field (e.g., Stefanatos et al. 1994; Helmis et al. 1995; Stefanatos et al. 1996). More recently, Tian et al. (2013) carried out wind-tunnel experiments to assess the effect of topography on the performance of an array of five wind turbines sited on a Gaussian hill. Moreover, Yang et al. (2015) performed wind-tunnel flow measurements for a wind turbine placed downwind of a sinusoidal hill and compared their LES results of the same case with experimental data, observing that the presence of the hill upstream of the turbine leads to a faster wake recovery. Furthermore, in their wind-tunnel study, Hyvärinen and Segalini (2017a) placed two turbines, one downstream of the other, on a series of periodic sinusoidal hills, whose heights were less than half the turbine hub-heights. They found that the presence of the hilly terrain produces a more rapid wake recovery of the first turbine and, hence, a higher power coefficient of the downwind turbine. Recently, a field measurement campaign in Perdigão, Portugal, was carried out to characterize the flow over a double-ridge complex terrain on which was sited a turbine (Mann et al. 2017; Fernando et al. 2018).

With regard to the numerical simulations of the flow past wind turbines in topography, previous research focused on finding numerical solutions of the problem (e.g., Voutsinas et al. 1990b; Hemon et al. 1991; Günther et al. 1993; Ansorge et al. 1994; Chaviaropoulos et al. 1999; Ivanova and Nadyozhina 2000; Migoya et al. 2007). More recently, Politis et al. (2012) simulated the flow in a real wind farm in Spain using a RANS approach and compared the power outputs with field measurements. They also studied the flow through a turbine sited on top of a single Gaussian hill, observing that the presence of the hill leads to a slower wake recovery. Moreover, using terrain-following coordinates, Shamsoddin and Porté-Agel (2017a) performed LES of flow through a wind farm sited on a single hill and validated the results using the dataset of Tian et al. (2013). By linearizing the continuity and momentum equations, Segalini (2017) developed a numerical model that accounts for both wind turbines and low-slope topography. The advantage of the model is its lower computational cost. Apart from these, other numerical studies have been carried out both for real complex terrain (Schulz et al. 2014; Yang et al. 2014c; Castellani et al. 2015, 2017; Berg et al. 2017; Wagner et al. 2019) and idealized hills (Yang et al. 2015; Zheng et al. 2017).

To model the effect of topography on the wind-turbine wakes with simple methods, many researchers have used the straightforward idea of simply superposing the turbine wake’s velocity deficit over flat terrain on the flow (without turbines) over the topography (e.g., Crespo and Hernández 1986; Crespo et al. 1993; Hyvärinen and Segalini 2017b). The superposition method is standard in industry and, although this method is deemed to yield acceptable predictions for moderate topography (Crespo et al. 1993; Hyvärinen and Segalini 2017b), its general applicability, even for hills with moderate slopes, is questionable (Politis et al. 2012; Segalini 2017).

Herein, we point out three key aspects of wake flows over topography that potentially contribute to inaccuracies of a superposition approach; namely, (i) non-zero pressure gradients, (ii) variable elevation of the wake-centre trajectory from the ground, and (iii) flow separation (or non-separated sheltering). The variations in the underlying terrain elevation lead to streamwise pressure gradients and, hence, to streamwise accelerations and decelerations in the flow (without turbines). For example, considering the flow over a single hill, we have favourable pressure gradient regime on the windward side of the hill and an adverse pressure gradient regime on the leeward side. It has been shown in the literature that the pressure gradient can noticeably affect the wake recovery in such a way that wakes recover faster under favorable pressure gradient and slower under adverse pressure gradient. For two-dimensional wakes, this phenomenon has been substantiated experimentally (Liu et al. 2002; Thomas and Liu 2004), numerically (Rogers 2002), and theoretically (Shamsoddin and Porté-Agel 2017b). Shamsoddin and Porté-Agel (2018a) recently demonstrated that the same trend holds for axisymmetric wakes. Moreover, they developed an analytical model to account for the effect of the pressure gradient on wake recovery and validated the model with a LES dataset. In this work, the effect of the pressure gradient is totally decoupled from the effect of streamline curvature. Therefore, it is shown that pressure gradient, alone, is responsible for this considerable change in the wake recovery. Another interesting point about the effect of pressure gradient is that it alters the rate of change of the wake-centre velocity deficit and wake width in different directions. For instance, as already mentioned, a favourable pressure gradient regime increases the wake-centre velocity recovery rate, but it decreases the wake-width growth rate. Bearing in mind that superposition methods do not consider the pressure gradient effect, these notions and models can be a valuable addition to the existing common practice.

The second and third aspects, i.e., variable elevation of the wake-centre trajectory and flow separation (or non-separated sheltering), are intertwined because the flow separation (or non-separated sheltering) in the leeward side of the hill deflects the wake-centre trajectory upwards. To acquire a clearer idea of the concept of non-separated sheltering, among others, see Belcher (1999). In fact, in superposition methods, the wake trajectory is simply shifted vertically with the same distance as the terrain elevation. Moreover, in these methods, the models that are used to calculate flow over the topography (without turbines) do not account for flow separation. These two problems lead to erroneous predictions of wake trajectory that, in turn, can lead to an inefficient design of wind farms sited on topography.

**Fig. 18:**
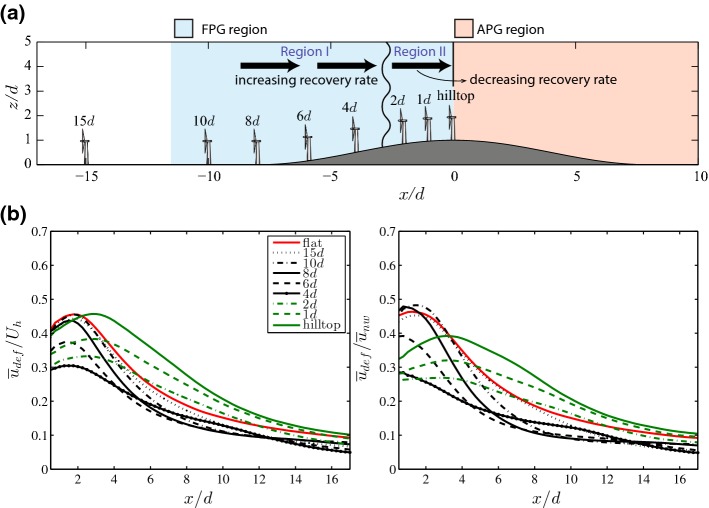
Effect of pressure gradient on the recovery of wind-turbine wakes. Figure reprinted from Shamsoddin and Porté-Agel (2018b) with the permission of Cambridge University Press. Note that *APG*$=$ adverse pressure gradient, *FPG*$=$ favourable pressure gradient. **a** Schematic of different turbine placements with respect to the hill. The distance of the turbine to the hilltop is shown on top of the turbine for each placement case. **b** Wake-centre velocity recovery for different turbine positions with respect to the hill. The velocity deficit $\overline{u}_{\mathrm {def}}$ is normalized in two ways: in the left panel, the undisturbed flow speed at hub-height $U_h$ is used, and in the right panel, the deficit at each point is normalized by the no-wake flow speed of exactly the same point $\overline{u}_{nw}$

More recently, Shamsoddin and Porté-Agel (2018b) developed an analytical modelling framework to model wake flows over two-dimensional hills. The model accounts for the effects of both the pressure gradient (as discussed above) and the hill-induced streamline distortion. Moreover, a special treatment is carried out for the behaviour of the wake on the leeward side of the hill. It is shown that the wake trajectory for a hill of the same height as the turbine hub-height follows the hill profile fairly closely in the windward side, but it maintains an almost constant elevation (a horizontal line) downstream of the hilltop. Furthermore, to show the effect of the hill-induced pressure gradient on the wind-turbine wake recovery from a more practical point of view, a parametric study of the position of the turbine with respect to the hill is performed (Fig. 18a, b). The important observation is that, when the turbine is moved from far upstream towards the hilltop, the wake recovery rate increases up until a certain distance to the hilltop (region I in Fig. 18a). After this point, if we move the turbine closer to the hilltop, the recovery rate decreases (region II). This is especially clear when the turbine is placed at the hilltop, where the wake recovery rate is significantly smaller due to the adverse pressure gradient on the leeward of the hill.

## Vertical-Axis Wind Turbines

In addition to horizontal-axis wind turbines, vertical-axis wind turbines (VAWTs) are alternative devices for wind-energy harvesting. Although these two types of machines have similarities in many aspects, there are some intrinsic differences between the two, making the separate study of VAWTs necessary. In general, VAWTs can be categorized into two types: the drag-driven type (e.g., the Savonius rotor) and the lift-driven type (e.g., the Darrieus rotor) (Paraschivoiu 2002). As the tip-speed ratio of drag-driven devices cannot exceed unity, their maximum power coefficient is much less than their lift-driven counterparts (values of approximately half the Betz limit have been reached in practice, e.g., Manwell et al. 2010). The maximum power coefficient of lift-driven VAWTs, however, reaches the Betz limit in ideal conditions, as is the case with HAWTs (Manwell et al. 2010). Herein, we only consider the development of lift-driven devices.

The concept of a lift-driven VAWT was first introduced by the patent of Darrieus (1931). Later in the 1970s and 1980s, the performance of VAWTs was investigated mainly by North American institutes, including the National Research Council of Canada, NASA Langley Research Center, and the Sandia National Laboratories (Blackwell et al. 1976; Sheldahl and Blackwell 1977; Sheldahl et al. 1980; Johnston 1980; Worstell 1981; McNerney 1981). Other researchers during this time period, mainly using wind-tunnel measurements, investigated VAWT performance (South and Rangi 1975; Nguyen et al. 1981; Vittecoq and Laneville 1982; Penna and Kuzina 1984; Schienbein 1979; Richards 1987), with the focus of all these studies mostly on the overall rotor performance (e.g., power and torque) and loading on the blades. On the other hand, the first measurements of the VAWT wakes can be traced back to Muraca and Guillotte (1976), Vermeulen et al. (1979), Strickland et al. (1981), Brochier et al. (1986) and Bergeles et al. (1991). More recently, and especially with the emergence of the particle-image velocimetry technique, more VAWT wake measurements have been made both in the near-wake (Battisti et al. 2011; Tescione et al. 2013; Bachant and Wosnik 2015; Araya and Dabiri 2015) and far-wake (Brochier et al. 1986; Rolin and Porté-Agel 2015; Ryan et al. 2016; Rolin and Porté-Agel 2018) regions.

Apart from experimental investigations, some analytical models have also been proposed for the prediction of the VAWT performance. One can categorize these models into two groups. The first group comprises the streamtube models, which are based on the principle of conservation of momentum. There are different kinds of such models (single-, double-, and multiple-streamtube models); however, the double-streamtube model has proven to attract more attention in the community (e.g., Jafari et al. 2018a, b). By using streamtube models, one can obtain information about the overall performance of the turbine (e.g., power, torque, loading on the blades). The second group comprises the vortex models, which are based on the vorticity equations. Of the different types of these models, one can mention the fixed-wake (Wilson and Walker 1981) and free-wake (Strickland et al. 1981) types. With vortex models, unlike the streamtube models, it is also possible to obtain insight into the near-wake of the turbine. For an in-depth description of these analytical models, the reader is referred to Paraschivoiu (2002) (Chapters 4 and 6).

Vertical-axis wind turbines have also been investigated with numerical simulations, using two main approaches to represent the wind turbines. The first approach is to resolve the blades of the VAWT rotor (i.e., taking into account the geometry of the blade airfoil in the computational mesh) and the boundary layer around them. This approach, which has been used for example in Castelli et al. (2011), Li et al. (2013), Marsh et al. (2015), Bremseth and Duraisamy (2016), Posa et al. (2016), and Ghasemian et al. (2017), is capable of providing much information about the loading on the blades, the region inside the rotor area and the near-wake. However, because of its high computational cost, it becomes unpractical for simulation of the far-wake and, especially, simulation of large wind farms. The second approach is to model the VAWT rotor using the actuator-type techniques. With this approach, one can overcome the shortcomings of the first approach, making simulations of VAWTs in the ABL and in large domains computationally affordable (e.g., Rajagopalan and Fanucci 1985; Rajagopalan et al. 1995; Shen et al. 2009; Shamsoddin and Porté-Agel 2014, 2016; Hezaveh et al. 2017; Abkar and Dabiri 2017).

In the remainder of this section, we elaborate on some features of VAWTs and their wakes that are unique to VAWTs and that differentiate them from HAWTs. First, we have a look at the energetic performance of VAWTs. For this purpose, we consider a typical MW-size VAWT whose capacity is in the order of 1 MW. Such a VAWT has a typical rotor diameter of around 50 m and a rotor height of around 100 m (see Project Éole in Templin and Rangi 1983). Shamsoddin and Porté-Agel (2016) characterized the energetic performance of such a turbine (operating in the ABL) by calculating the power coefficient of the turbine for more than 100 different combinations of tip-speed ratios and blade chord lengths (i.e., different solidities). The optimum combination of solidity (defined as *Nc* / *R*, where *N* is the number of blades, *c* is the chord length, and *R* is the rotor radius) and tip-speed ratio is found to be 0.18 and 4.5, respectively. This combination results in a power coefficient of 0.47.

**Fig. 19:**
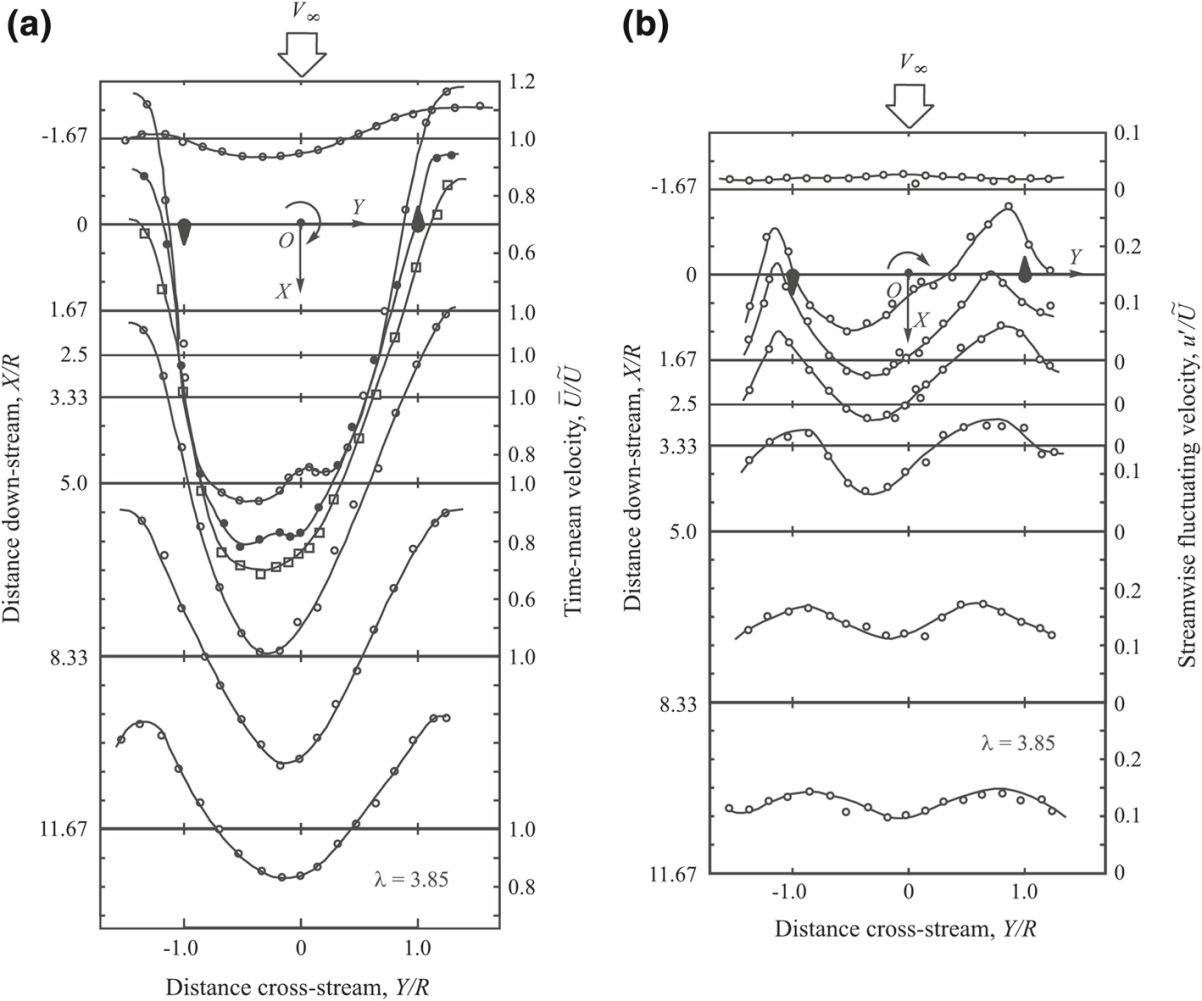
Wake of a VAWT with a tip-speed ratio of 3.85 measured in a water-channel experiment. **a** Mean velocity horizontal profiles (normalized by the inflow velocity). **b** Turbulence intensity profiles. In both figures the streamwise distance *X* is normalized by the rotor radius *R* Figure reprinted from Brochier et al. (1986) with the permission of The American Institute of Aeronautics and Astronautics (AIAA)

Another feature of VAWTs is that, unlike HAWTs, their wakes have asymmetries in the spanwise profiles of both the mean flow speed and the turbulence intensity (see Fig. 19). Regarding the mean flow speed, it has been demonstrated that the position of the maximum flow speed point shifts towards the windward side (i.e., the side where the streamwise components of the blade velocity and the incoming flow velocity have opposite directions) of the turbine. This is obviously because on the windward side the magnitude of the relative flow speed, with respect to the blades, is more than the leeward side (i.e., the side where the streamwise components of the blade velocity and the incoming flow velocity have the same direction), and consequently, the magnitude of the exerted force of the blades on the flow is higher. Concerning the turbulence intensity profile, the observations are less straightforward to interpret. The horizontal turbulence intensity profile has two local peaks, one on the windward side and one on the leeward side. In most of the experiments (e.g., Brochier et al. 1986; Bachant and Wosnik 2015; Posa et al. 2016), it is shown that the leeward peak has a higher value than the windward one. This was attributed to the stall vortices on the leeward side, despite the fact that the shear is the highest in the windward side due to the aforementioned asymmetry in the mean velocity profile. This is, however, in contrast with the measurements of Rolin and Porté-Agel (2015), where the windward peak was shown to have a higher value; this can be due to the relatively higher solidity of the model turbine in this experiment (Posa et al. 2016). The phenomenon of stall vortices can potentially be captured in numerical simulations of the blade-resolving type (e.g., Posa et al. 2016). However, as in the actuator-type simulations the stall (and consequently flow separation from the blades) cannot be captured, the higher turbulence intensity occurs at the windward side because of the higher shear on that side. It is also noteworthy that these asymmetries significantly depend on the tip-speed ratio of the turbine, such that with increasing the tip-speed ratio these asymmetries decrease; for example, a relatively small asymmetry is reported at tip-speed ratio of 4.5 in Shamsoddin and Porté-Agel (2016).

The general horizontal asymmetry of the flow field around VAWTs leads to interesting properties of these kinds of machines. It has been shown that placing two counter-rotating VAWTs side-by-side increases the total power efficiency of the pair (Dabiri 2011; Zanforlin and Nishino 2016).

## Summary

We have reviewed the relevant literature on experimental, computational, and theoretical studies of the interactions of ABL flow with wind turbines and wind farms. Emphasis has been placed on the current state of our understanding and ability to model wind-turbine wake flows and their impact on ABL structure and wind-farm performance. This knowledge is essential for optimizing the design and control of wind farms.

First, we have focused on the simplest case of the interaction between a stand-alone horizontal-axis wind turbine and the ABL over homogeneous flat terrain. The structure and dynamics of the main flow regions (induction, near-wake, and far-wake regions) are discussed, with emphasis on the role of atmospheric turbulence. The main conclusions can be summarized as follows:

- The near-wake region, whose structure and dynamics (e.g., tip and hub vortices) are affected by the geometry and operation of the wind turbine, has a length of about two to four rotor diameters, depending on the turbulence intensity in the ABL.
- The mean flow velocity in the far-wake region, which depends only on global turbine performance parameters (mainly $C_T$) and atmospheric turbulence, can be analytically modelled using conservation of mass and momentum, together with the assumptions of a Gaussian distribution of the velocity deficit and a nearly-linear wake expansion.
- Recent attempts have been made to estimate the role of atmospheric turbulence on the growth rate of the far-wake by using empirical relations as well as theoretical developments based on the analogy with passive scalar plumes.
- The above-mentioned analytical framework for the far-wake flow has been extended to the case of turbines working under yawed conditions by using conservation of momentum in both the streamwise and spanwise direction. Experimental and analytical evidence suggests that yawing can be used as an effective wake mitigation strategy.
- Meandering of the far-wake has been associated with the dynamics of relatively large (larger than twice the rotor diameter) turbulent eddy motions in the ABL. This connection has been used to develop models for the position of the instantaneous wake centre and the unsteady loads on downwind turbines.

Next, we have shifted our attention to the more complex case of the interaction between wind farms and the ABL. In this case, the superposition of multiple wind turbine wakes and their two-way interaction with the ABL flow can lead to substantial changes in both the structure of the ABL and the energetic efficiency of the farm. Different flow regions have been identified and studied for the case of flat homogeneous terrain: wind-farm induction region, development region, fully-developed region (where the flow is fully adjusted to the wind farm), and wind-farm wake region. The main conclusions can be summarized as follows:

- The extent of the different wind-farm flow regions is affected by the wind-farm characteristics (e.g., size and layout), as well as the thermal stability of both the surface layer and the free atmosphere. The latter can trigger standing gravity waves and flow ‘choking’ under subcritical flow conditions.
- Fully-developed wind-farm boundary-layer flow is only achieved after a very long distance (up to two orders of magnitude of the boundary layer height), which depends on factors such as free-atmosphere stratification and wind farm layout.
- One-dimensional models have been developed to predict area-averaged flow characteristics and wind farm performance (power output) in fully-developed wind-farm flows. These models have been used to parametrize the effects of very large wind farms in weather and climate models.
- In finite-size wind farms, turbine energetic performance is strongly affected by the wind direction, which effectively changes the farm layout with respect to the incoming wind. Analytical models of wake effects in wind farms have been developed based on the superposition of the aforementioned analytical single-wake models.
- Thermal stability has been shown to have a strong effect on wind-farm performance, as well as land–atmosphere exchanges of momentum and scalars.
- The non-stationarity of the ABL flow through the diurnal cycle leads to large variations in wind-farm flows and, consequently, power output. Particularly relevant at night are the effects of the LLJ and the vertical changes of wind direction associated with the Coriolis force.

Lastly, we have reviewed two research topics that have benefited from relatively little research so far: (*a*) vertical-axis wind turbines (VAWTs), their performance and their wakes; and (*b*) topography and its effects on the interaction of wind turbines with the ABL. These are the main conclusions:

- It is shown that the power coefficient of typical MW-size VAWTs can reach values as large as 0.47.
- It is shown that VAWT wakes can potentially have significant spanwise asymmetries in mean velocity and turbulence intensity profiles, which can lead to innovative turbine placement strategies in VAWT farms to maximize the wind farm efficiency.
- When turbines are sited on topography, the underlying terrain can have significant effects on both the trajectory and recovery of the wakes. Recent efforts have been made to quantify and analytically model these effects.
- Pressure gradients in the ABL, induced for example by topography, affect turbine wake recovery. Specifically, favourable pressure gradients (e.g., on the windward side of a hill) increase wake recovery, while adverse pressure gradients (e.g., on the leeward side of a hill) decrease it.

## Future Perspectives

Despite the remarkable progress made so far in the understanding and modelling of wind-turbine and wind-farm flows, many research questions remain unanswered or underexplored. Below, we provide a list of some possible future research directions.

- Extending the above-described analytical wake modelling framework to turbulence quantities (e.g., turbulence intensity and turbulent fluxes), so that it can be used to improve the prediction of both power losses and fatigue loads in wind farms.
- Developing a physics-based ‘theory’ for the superposition of multiple wakes in turbulent boundary layer flows.
- Further investigating the role of the different scales of atmospheric turbulence on wind turbine wake structure and dynamics for both stand-alone turbines and wind farms.
- Further understanding the effects of thermal stability on the interactions between the ABL and wind farms of different sizes and shapes.
- Developing improved, computationally efficient models of finite-size wind farms capable of capturing the two-way coupling and joint evolution of multiple turbine-wake flows and the overlying ABL flow.
- Developing and validating multi-scale simulation strategies for the prediction of the entire range of scales of interest in wind-farm aerodynamics. Particularly challenging is the coupling of coarse-resolution RANS-based weather models with high-resolution models, such as LES.
- Extending the study and modelling of topography effects to include complex (multi-scale) topography and thermal stratification effects.
- Investigating alternative wind-turbine technologies, including for example different types of VAWTs and multi-rotor (horizontal-axis and vertical-axis) turbines, as well as their wind farms.
- Developing and testing improved wind-farm control strategies to maximize overall wind-farm performance via wake mitigation maneuvers, such as yawing and/or downregulation, applied to selected groups of wind turbines.
- Further investigating and developing models for wind-farm wake flows and their effect on the performance of neighboring wind farms in regions of high wind-energy penetration, such as the North Sea.
- Further investigating the effect of wind farms on land-atmosphere exchanges, as well as the potential impact of land-use and climate change on wind-energy potential.
- Designing and performing high-quality wind-tunnel and field experiments to provide further physical insight on the flow, and to guide the improvement, calibration and validation of the aforementioned numerical models.
- Using recent advances in wind-turbine and wind-farm aerodynamics in support of other wind-energy research areas not covered in this article. These include aerodynamic noise, structural health, and wind-farm operation aspects such as control, sensing, diagnostics, monitoring, energy storage, and grid integration. A recent review of these topics is given by Willis et al. (2018). Another review of long-term multidisciplinary research challenges in wind energy is given by van Kuik et al. (2016).
